# Impairing Gasdermin D-mediated pyroptosis is protective against retinal degeneration

**DOI:** 10.1186/s12974-023-02927-2

**Published:** 2023-10-20

**Authors:** Rakshanya Sekar, Yvette Wooff, Adrian V. Cioanca, Melan Kurera, Chinh Ngo, Si Ming Man, Riccardo Natoli

**Affiliations:** 1 The John Curtin School of Medical Research, The Australian National University, Canberra, ACT Australia; 2 School of Medicine and Psychology, The Australian National University, Canberra, ACT Australia

**Keywords:** Gasdermin D, Age-related macular degeneration (AMD), Retinal degenerations, Neuroinflammation, Inflammasome, IL-1β, Extracellular vesicles

## Abstract

**Background:**

Inflammasome activation and the subsequent release of pro-inflammatory cytokines including Interleukin 1β (IL-1β) have been widely reported to contribute to the progression of retinal degenerations, including age-related macular degeneration (AMD), the leading cause of blindness in the Western World. The role of Gasdermin D (GSDMD), a key executioner of pyroptosis following inflammasome activation, however, is less well-established. In this study we aimed to characterise the role of GSDMD in the healthy and degenerating retina, and uncover its role as a conduit for IL-1β release, including via extracellular vesicle (EV)-mediated release.

**Methods:**

GSDMD mutant and knockout mice, in vitro models of inflammation and a well-established in vivo model of retinal degeneration (photo-oxidative damage; PD) were utilised to explore the role and pathological contribution of GSDMD in regulating IL-1β release and propagating retinal inflammation. RNA sequencing of whole retinas was used to investigate GSDMD-mediated inflammation during degeneration. The role of EVs in GSDMD-mediated IL-1β release was investigated using nanoparticle tracking analysis, ELISA and EV inhibition paradigms. Finally, the therapeutic efficacy of targeting GSDMD was examined using GSDMD-specific siRNA.

**Results:**

We identified in this work that mice deficient in GSDMD had better-preserved retinal function, increased photoreceptor survivability and reduced inflammation. RNA-Seq analysis revealed that GSDMD may propagate inflammation in the retina via NF-κB signalling cascades and release of pro-inflammatory cytokines. We also showed that IL-1β was packaged and released via EV in a GSDMD-dependent manner. Finally, we demonstrated that impairing GSDMD function using RNAi or blocking EV release was able to reduce IL-1β content in cell-free supernatant and EV.

**Conclusions:**

Taken together, these results suggest that pyroptotic pore-forming protein GSDMD plays a key role in the propagation of retinal inflammation, in particular via the release of EV-encapsulated IL-1β. Targeting GSDMD using genetic or pharmacological inhibitors may pose a therapeutic opportunity to dampen inflammatory cascades and delay the progression of retinal degeneration.

**Supplementary Information:**

The online version contains supplementary material available at 10.1186/s12974-023-02927-2.

## Background

Age-related macular degeneration (AMD) is the leading cause of blindness in the developed world and is characterised by the death of the light-sensing photoreceptor cells and underlying retinal pigmented epithelium (RPE) in the central retina, leading to permanent and irreversible vision loss [1]. Chronic inflammation is a key driver in the onset and progression of AMD, with the role of the inflammasome, a multimeric protein complex of the innate immune system, well-established in the pathogenesis of this disease [2–5]. While large bodies of evidence support the role of various inflammasome components, such as NLRP3, Caspase-1 (CASP-1), and Interleukin-1β (IL-1β) in AMD pathogenesis [5–8], the role of terminal inflammasome component pyroptotic pore-forming protein Gasdermin D (GSDMD) in the retina is currently not well-established.

GSDMD forms pores on the cell surface following activation of the inflammasome in response to inflammatory priming signals [6, 9]. GSDMD consists of an N-terminal (NT) pore-forming domain and a C-terminal (CT) autoinhibitory domain, with a cleavage site in the linker between at position D276 (in mouse) and D275 (in human) [10, 11]. Upon cleavage by inflammasome effector protein Caspase-1 or Caspase-11, GSDMD-NT mobilises from the auto-inhibitory complex in the cytoplasm and inserts into the inner leaflet of the plasma membrane, inducing the formation of ~ 21 nm diameter membrane pores [12–14]. Membrane pore formation allows for passive transport of molecules, including ions, resulting in loss of osmotic homeostasis, cell swelling, and a lytic form of cell death known as pyroptosis. Importantly, the activation and formation of GSDMD pores on the cell surface induces the release of pro-inflammatory mediators, including cytokines Interleukin-18 (IL-18) and IL-1β [15, 16]. As the activation and release of mature IL-1β has well-established roles in initiating and propagating inflammation in AMD [5, 8, 17, 18], including in macrophage recruitment [19], modulating chemokine expression [8], and in the activation of other pro-inflammatory cytokines, such as IL-6 [20]; understanding the role that GSDMD plays in the degenerating retina and how IL-1β is released and propagated far from the initial damage site is a key strategy in combating retinal inflammation, and slowing disease progression.

Recent evidence has suggested that IL-1β may be released encapsulated in extracellular vesicles (EVs) in a GSDMD-dependant manner [21, 22], in response to calcium influx during membrane pore formation [23–25]. In addition, it has been proposed that GSDMD may promote EV release at the site of pore-assembly to enable membrane repair and return to homeostasis [14, 26]. EVs are lipid–membrane enclosed nanoparticles that facilitate cell to cell communication via the transport of molecular cargo [27]. Increased EVs have been reported in inflammatory and neurodegenerative diseases, functioning to propagate inflammatory cascades and growing evidence indicates their potential role in disease pathogenesis [28–30]. However, to date, there have been only a few studies to investigate GSDMD in the retina, and little understanding on GSDMD-mediated EV release or its role in IL-1β transport as a means to propagate inflammation.

The few studies investigating the role of GSDMD in the retina have predominantly utilised cell-culture-based techniques and focused only on the RPE using amyloid beta (Aβ) (a toxic component known to accumulate in dry-AMD) treated aRPE-19 cells [31, 32] or Alu RNA (a transposon group implicated in dry-AMD) transfected human RPE cells [7, 33]. In-vivo, upregulation of GSDMD-NT in the RPE cells of Long-Evans rats following Aβ intravitreal injections in a model of dry-AMD resulted in swollen RPE cells and elevated IL-1β production [34]. This suggests GSDMD may be a key player in the release of IL-1β and subsequent RPE degeneration during AMD. Investigations into the role of GSDMD in the retina, including characterising protein expression and release of pro-inflammatory cytokines, is, therefore, necessary to understand its contribution to chronic inflammation in retinal degenerations, including AMD. Furthermore, as the release point for pro-inflammatory cytokines, impairing GSDMD-mediated pore formation and subsequent pyroptosis presents a therapeutic opportunity, given the strong association between pro-inflammatory cytokine IL-1β and AMD disease pathogenesis [8, 17, 35]. While, IL-1β inhibition through small interfering RNA (siRNA) or neutralizing antibodies has previously demonstrated therapeutic efficacy in models of retinal degenerations, reducing immune cell recruitment to the outer retina and decreasing subsequent production of chemokines from Müller glia and RPE cells [4, 8], pro-active mitigation of inflammation by preventing the release of mature IL-1β and other pro-inflammatory cytokines by targeting GSDMD represents a novel approach to tackle inflammation.

This study, therefore, aims to investigate the role of GSDMD in the retina during health and degeneration using an in-vivo photo-oxidative damage (PD)-induced model of retinal degeneration [36]. We have previously shown that mice undergoing PD have increased levels of oxidative stress and inflammation, initiating pathological changes, such as microglia activation and recruitment, and focal photoreceptor cell loss, as seen in AMD [35, 36]. In this study we have demonstrated that GSDMD is primarily expressed in subretinal macrophages of PD mice and has increased gene and protein expression across PD. Moreover, mice with impaired GSDMD function (*Gsdmd*^*I105N/I105N*^* mice*) displayed preserved retinal function, increased photoreceptor survivability and reduced inflammation following 5-day PD. RNA-Seq data supports a mechanism of NF-κB-mediated inflammation resulting in GSDMD-induced release of pro-inflammatory cytokines. Finally, we show that GSDMD may mediate inflammation via packaging and release of pro-inflammatory cytokines, such as IL-1β via EVs in addition to release through transmembrane pores or after membrane rupture, and that impairing GSDMD function using RNAi or blocking EV release in pyroptotic cells may pose a therapeutic opportunity for reducing IL-1β release and targeting inflammation in retinal degeneration.

## Methods

### Animal handling and photo-oxidative damage

All experiments were conducted in accordance with the ARVO Statement for the Use of Animals in Ophthalmic and Vision Research and with approval from the Australian National University’s (ANU) Animal Ethics Committee (AEC) (Ethics ID: A2020/41). Adult male and female C57BL/6J wild type (WT), Gasdermin D mutant *Gsdmd*^*I105N/I105N*^ and Gasdermin D knockout *Gsdmd*^*−/−*^ mice (aged 60–80 postnatal days) were bred and reared under 12 h light (5 lux)/dark cycle conditions with free access to food and water. *Gsdmd*^*I105N/I105N*^ mice were generated at the ANU and *Gsdmd*^*−/−*^ mice were generated at Genentech, with both mouse strains described in detail by Kayagaki, N et al. [37]. Age-matched and sex-matched mice were randomly assigned to 5-day photo-oxidative damage (PD) or dim-reared control (DR) groups (*N = *4–7 per group). Animals in the photo-oxidative damage group were exposed to 100,000 lux white LED light for a period of 5 days and were administered a pupil dilator (Minims^®^ atropine sulphate 1% w/v; Bausch and Lomb) to both eyes twice a day (9am and 4 pm) during the damage paradigm [36]. Dim-reared mice were maintained in standard housing conditions of 12 h light (5 lux)/dark cycle with free access to food and water.

### Retinal assessment

#### Retinal function via electroretinography (ERG)

Full-field scotopic electroretinography (ERG) was performed to assess the retinal function of WT, *Gsdmd*^*I105N/I105N*^ and *Gsdmd*^*−/−*^ mice in DR conditions or following PD. Mice were dark-adapted overnight before being anaesthetised with an intraperitoneal injection of Ketamine (100 mg/kg; Troy Laboratories, NSW, Australia) and Xylazil (6 mg/kg; Troy Laboratories, NSW, Australia) diluted in PBS. Both pupils were dilated with one drop each of 2.5% w/v phenylephrine hydrochloride and 1% w/v tropicamide (Bausch and Lomb, NY, USA).

Anaesthetised and pupil dilated mice (as above) were placed on the thermally regulated stage of the Celeris ERG system (Diagnosys LLC, MA, USA) [38]. The Celeris ERG system has combined Ag/AgCl electrode–stimulator eye probes which measure the response from both eyes simultaneously and uses 32-bit ultra-low-noise amplifiers fitted with impedance testing. Eye probes were cleaned with 70% ethanol, and then a Hypromellose 0.3% eye drop solution (GenTeal; Novartis, NSW, Australia) was applied to both probes. The probes were then placed covering and just touching the surface of each eye. A single-flash paradigm was used to elicit a mixed response from rods and cones. Flash stimuli for mixed responses were provided using 6500 K white flash luminance range over stimulus intensities from − 2.0 to 1.6 log cd s m^−2^. Interstimulus interval was increased from 2 s for the lowest intensities (steps 1–4) to 10 s for steps 4–5 and up to 1 min (steps 5–6) for the highest intensity to allow complete recovery of the b-wave between stimuli. Responses were recorded and analysed using Espion V6 Software, (Diagnosys LLC, MA, USA). Statistics were performed in Prism V9.4 (GraphPad Software, CA, USA) using a two-way analysis of variance (ANOVA) to test for differences in a-wave and b-wave responses. Data were expressed as the mean wave amplitude ± SEM.

### Retinal tissue collection and preparation

Animals were euthanised with CO_2_ following functional ERG analysis. The superior surface of the right eye from each animal was marked and enucleated, then immersed in 4% paraformaldehyde (PFA) in PBS for 3 h at 4 °C. Eyes were then cryopreserved in 15% sucrose solution overnight, embedded in OCT medium (Tissue Tek, Sakura, JP) and cryosectioned at 12 μm in a parasagittal plane (superior to inferior) using a CM 1850 Cryostat (Leica Biosystems, Germany). To ensure accurate comparisons were made for histological analysis, only sections containing the optic nerve head were used for analysis. The retina from the left eye of each mouse was excised through a corneal incision and placed into RNAlater solution (Thermo Fisher Scientific, MA, USA) at 4 °C overnight and then stored at − 80 °C until further use [39].

### Immunolabelling

Immunohistochemical analysis of retinal cryosections was performed as previously described [36]. Fluorescence was visualised and images obtained using a laser-scanning A1 + confocal microscope at 20× and 40× magnification (Nikon, Tokyo, Japan). Images panels were analysed using ImageJ V2.0 software (NIH, MD, USA) and assembled using 27.0.1 Illustrator software (Adobe Systems, CA, USA).

#### IBA-1 immunohistochemistry

Immunolabeling for IBA-1 (1:1000, Anti-Iba1, Rabbit, 019-19741, Wako, Osaka, Japan or 1:1000, Anti-Iba1, Rat, ab283346, Abcam, United States) and quantification was performed as previously described [36]. The number of IBA-1^+^ cells (a marker of retinal microglia and macrophages) was counted across the superior and inferior retina using two retinal sections per mouse and then averaged. Retinal cryosections were stained with the DNA-specific dye bisbenzimide (1:10,000, Sigma-Aldrich, MO, United States) to visualize the cellular layers. Images of IBA-1 + staining were captured with the A1 + confocal microscope at 20× magnification.

#### Gasdermin D immunohistochemistry

Immunolabeling for GSDMD and quantification was performed on optic nerve cross sections. Cryosections were incubated in 10% normal goat serum (I5256-10MG, Sigma-Aldrich) for 1 h at room temperature (RT) followed by incubation in GSDMD primary antibody (1:1000, rabbit monoclonal, ab209845, Abcam, United States) overnight at 4 °C. The following day, sections were washed in Ultrapure Endotoxin-free 0.1 M PBS (Thermo Fisher Scientific, MA, USA) and incubated in Alexa Fluor™ 488 secondary antibody (1:5000, Goat anti-Rabbit IgG, Invitrogen) for 2 h at RT. The sections were washed in 1 × PBS thrice (15 min per wash) and then stained (1:10,000 bisbenzimide, Sigma-Aldrich, MO, United States) to visualize the cellular layers, and cover slipped with aqueous mounting medium (Aquamount, cat. no. 18606; Polysciences, Warrington, PA). Immunofluorescence was viewed and captured with the A1 + confocal microscope at 20× and 40× magnification. Alexa Fluor™ 488 (detected in green channel) was pseudo-labelled as yellow LUT using ImageJ V2.0 software (NIH, MD, USA) for visualisation.

#### TUNEL assay

Terminal deoxynucleotidyl transferase (Tdt) dUTP nick end labelling (TUNEL) was used as a measure of photoreceptor cell death. TUNEL in situ labelling was performed on retinal cryosections using a Tdt enzyme (Cat #3,333,566,001, Sigma-Aldrich, MO, USA) and biotinylated deoxyuridine triphosphate (dUTP) (Cat #11,093,070,910, Sigma-Aldrich, MO, USA) as previously described [36]. Images of TUNEL staining were captured with the A1 + confocal microscope at 20× magnification.

#### Histological analysis

To determine photoreceptor cell death, the total number of TUNEL^+^ cells were counted in each retinal section, including both the superior and inferior retina. This process of quantification was performed on two retinal sections per animal and was calculated as the average number of TUNEL^+^ cells per retinal section. Images of TUNEL staining were captured with the A1+ confocal microscope at 20× magnification. The thickness of the outer nuclear layer (ONL) was determined by counting the number of rows of nuclei (photoreceptor cell bodies) in the area of retinal lesion development (1 mm superior to the optic nerve head), to quantify photoreceptor survival. The process of ONL photoreceptor cell row quantification was performed five times per retina, on two retinal sections at comparable locations per mouse.

As a measure of inflammation, the number of IBA-1^+^ cells (a marker of retinal microglia and macrophages) were counted across the superior and inferior retina in retinal cryosections. This quantification was performed on two retinal sections per mouse and averaged. Bisbenzimide staining was used to visualise the cellular layers.

### Gene expression analysis

#### RNA extraction

Total RNA was extracted from the retinal samples using a mirVana™ Total RNA Isolation Kit (Thermo Fisher Scientific, MA, USA) according to the manufacturer’s instructions. The concentration and quality of each RNA sample was assessed using the Agilent 2100 Bioanalyzer.

#### cDNA synthesis and quantitative real-time polymerase chain reaction

Following RNA extraction, cDNA was synthesised from 1 µg of each RNA sample using a Tetro cDNA Synthesis Kit (Bioline, London, UK) according to the manufacturer’s protocol. Gene expression was measured using qRT-PCR using both mouse- and human-specific TaqMan hydrolysis probes (Thermo Fisher Scientific, MA, USA), as shown in Table 1. The TaqMan probes, cDNA and TaqMan Gene Expression Master Mix (Thermo Fisher Scientific, MA, USA) were plated in a 384-well transparent plate. Each reaction was performed in technical duplicate and was carried out using a QuantStudio 12 K Flex RT-PCR machine (Thermo Fisher Scientific, MA, USA). Analysis was performed using the comparative Ct method (ΔΔCt). Results were analysed as a percent change relative to control samples and normalised to reference gene glyceraldehyde-3-phosphate dehydrogenase (Gapdh).

**Table 1:** TaqMan probes (Thermo Fisher Scientific, MA, USA) used for qPCR

Gene symbols	Gene name	Catalogue number
*Asc/Pycard*	PYD and CARD domain containing	Mm00445747_g1
*Casp-1*	Caspase-1	Mm00438023_m1
*Ccl2*	Chemokine (C–C motif) ligand 2	Mm99999056_m1
*C3*	Complement component C3	Mm00437858_m1
*Gapdh*	Glyceraldehyde-3-phosphatase dehydrogenase	Mm01536933_m1
*Il-1β*	Interleukin-1β	Mm00434226_m1
*Il-6*	Interleukin-6	Mm00446190_m1
*Il-18*	Interleukin-18	Mm00434226_m1

#### High-throughput RNA sequencing (HTS) and bioinformatics

Following RNA extraction as described above, RNA was dried in stabilisation tubes overnight (NovogeneAIT Genomics Singapore Pte, Singapore) and shipped to NovogeneAIT Genomics Singapore for bulk RNA sequencing. Sequencing libraries were constructed with the Illumina TruSeq unstranded RNA library preparation kit using polyA selection for mRNA enrichment, then sequenced on the Illumina NovaSeq6000 platform acquiring ~ 20 million, 150 base-pair, paired-end reads per sample. Read phred scores, adapter/index contamination were checked with FastQC (Babraham Bioinformatics), then aligned to mouse genome (mm39) using Rsubread aligner with default parameters [40]. Alignments were summarised with featureCounts, genes with low expression (< 1 count per million) were filtered out, and normalisation factors for remaining genes were calculated using trimmed means of m (TMM) method [41]. Normalised counts were prepared for linear modelling using voom transformation [42] and then a statistical model was fitted using lmFit [43] and moderated t-statistics were computed using eBayes function. Tables of fold change and *p* value estimates were generated with topTable function and genes with adjusted *p* value < 0.1 were deemed differentially expressed. Similarity matrices used for hierarchical clustering were computed using Euclidean distance calculations, then sample agglomeration was performed using average linkage. Gene ontology analysis was performed using Enrichr. Sample clustering was assessed using principal component analysis and hierarchical clustering employing single linkage for sample agglomeration and Euclidean distance as measure of similarity (prcomp and hclust base R functions). Pathway analysis and functional clustering was carried out using gene set enrichment analysis (GSEA) with the M2 Molecular Signature Database, M5 Ontology gene sets, C5 ontology gene sets and MH mouse-ortholog hallmark gene sets [44]. To determine potential cellular locations of the differentially expressed genes, filtered single cell RNA sequencing data were downloaded from GSE153674 [44], comprising 4659 cells isolated from heathy, C57BL/6J mouse retina. The count matrix was processed using the following sequence of commands in Seurat 4.1: SCTransfrom() > RunPCA() > RunUMAP() > FindNeighbors() > FindClusters(). Cell types were assigned to each cluster using scType [45], then module score expression was calculated for each cell type in the downregulated (logFC < 1) and upregulated (logFC > 1) list using AddModuleScore () function in Seurat.

### In vitro experiments

#### Bone-marrow-derived macrophage (BMDM) extraction and differentiation

Primary BMDM were extracted from C57BL/6J WT, *Gsdmd*^*I105N/I105N*^ and *Gsdmd*^*−/−*^ mice as per previously described methods [46]. The ends of the tibia and femur were each cut, and the bones were held vertically over a 50 mL Falcon^TM^ tube. Marrow was flushed from the bones using 10 mL of BMDM media in a sterile 10 mL syringe with a 25-gauge needle (Becton Dickinson, NJ, USA). Once all marrow was collected, marrow was resuspended in BMDM media using a 27-gauge needle (Becton Dickinson, NJ, USA). Marrow samples were topped up to 30 mL media total volume and shared evenly between three non-treated 15 cm tissue culture dishes (Corning, Sigma-Aldrich), and grown at 37 °C in a humidified incubator supplied with 95% air and 5% CO2. 5 ml BMDM media was added to each dish after 3-day incubation. BMDM were ready for harvesting or stimulation at day 5. To harvest BMDM, cells were scraped off culture dishes with 23 cm cell scrapers (Thermo Fischer Scientific, MA, USA), spun down at 900 g for 5 min and re-seeded at a density of 2.0 × 10^6^ cells/well into 6 well plates (Nunc, Thermo Fisher Scientific, MA, USA).

#### Immortalised BMDM (iBMDM)

Immortalised BMDM were maintained in complete DMEM media (DMEM; Sigma-Aldrich, 10% FBS; Sigma-Aldrich, 1X Antibiotic–Antimycotic; Thermo Fisher Scientific, MA, USA) and seeded at a density of 1.0 × 10^6^ cells/well into 6 well plates (Nunc, Thermo Fisher Scientific, MA, USA) before commencing experiments.

#### In vitro inflammasome stimulation

24 h before in vitro stimulation, the cell culture media was changed from DMEM + 10% FBS to DMEM + 10% EV depleted FBS. Post-acclimatisation in media, BMDM from WT, *Gsdmd*^*I105N/I105N*^ and *Gsdmd*^*−/−*^ mice or iBMDM were primed with 20 ng/mL LPS from *Escherichia coli* 0111: B4 (N4391, Sigma Aldrich, MO, USA) for 4 h and stimulated with 5 mM ATP (A6419, Sigma Aldrich, MO, USA) for 0.5 h. Unstimulated cells were used as negative controls. For extracellular vesicle (EV) isolation supernatants were collected immediately from both control and LPS/ATP groups. Following stimulation, the media was changed, and cells were left for 24 h for further EV secretion, and supernatant collected. The supernatant was also used for IL-1β protein quantification experiments.

#### In vitro EV inhibition

EV inhibition on iBMDM was performed using GW4869 (Sigma-Aldrich, MO, United States), a known inhibitor of exosome biogenesis and release. GW4869 was reconstituted in dimethyl sulfoxide (DMSO; Sigma-Aldrich, MO, United States) to a concentration of 5 mM and used as a stock solution for further dilution in Ultrapure Endotoxin-free 0.1 M PBS. iBMDM were treated with 10 μM of GW4869 for 4 h before inflammasome stimulation.

#### In vitro* Gsdmd* inhibition

*Gsdmd* Silencer^®^ Select siRNA (Thermo Fisher Scientific, MA, USA) was encapsulated in Lipofectamine^®^ RNAiMAX (#13778-150, Invitrogen) following the manufacturer’s protocol (25 pmol/1.0 × 10^6^ cells/well). After 6 h, iBMDM underwent in vitro inflammasome stimulation.

### EV isolation

EVs from *Gsdmd*^*I105N/I105N*^***,**** Gsdmd*^*−/−*^, WT BMDM and iBMDM cell culture supernatants were isolated using differential ultracentrifugation. Cell culture supernatant were spun at 3200×*g* for 10 min at 4 °C to remove cells and cell debris. The supernatant was transferred to 14 × 89 mm Beckman Ultra-Clear ultracentrifuge tubes and centrifuged at 10,000×*g* for 30 min (Beckman Coulter, CA USA) using a Beckman Coulter Optima XE-100 ultracentrifuge fitted with a SW41Ti Rotor (Beckman Coulter, CA USA), to collect large EV. Large EV pellets were resuspended in 20uL Ultrapure Endotoxin-free 0.1 M PBS (Thermo Fisher Scientific, MA, USA). The EV-containing supernatant was transferred to a new ultracentrifuge tube and spun at 100,000 ×*g* for 90 min at 4 °C. The supernatant was decanted, and EV pellets were resuspended via titration for 1 min in 20 μL Ultrapure Endotoxin-free 0.1 M PBS (Thermo Fisher Scientific, MA, USA) and stored in − 80 °C until quantification.

### EV characterisation

The size and concentration of cell culture EV were measured using Nanoparticle Tracking Analysis on a NanoSight NS300 (Malvern Instruments, Malvern, United Kingdom). BMDM EV samples were diluted 1:50 in 1 ml Ultrapure Endotoxin-free 0.1 M PBS to achieve a particle per frame value between 40 and 100. Samples were analysed under constant flow provided by a syringe pump set at a speed of 35 (equating to ∼ 3.1 μl/min; Gerritzen et al., 2017). A total of nine 30 s long videos were captured (camera setting: 14) for each sample. The detection threshold was set between 4 and 5 and was not altered between measurements of the same experiment. The concentration values, modal, and mean sizes were exported to Prism V9.2 (GraphPad Software, CA, United States) for statistical analysis and plotting.

### Western blot

Western blot was used to measure the protein expression of GSDMD in BMDM stimulated with LPS/ATP (as described above), DR, and 5d PD retinal tissue lysate. The blot was performed as previously described (Jiao et al., 2018). 20 µg of protein was loaded into a 4–20% or 4–12% Mini-Protean TGX Precast Protein gel (Bio-Rad, CA, USA and subjected to electrophoresis (60 min, 100 V). The protein bands were transferred (45 min, 30 V) to a nitrocellulose membrane (Bio-Rad, CA, United States) using the Power Blotter semi-dry system (Thermo Fisher Scientific, MA, United States). Membranes were then washed in PBS-Tween (0.05%; PBS-T), blocked in 5% BSA for 1 h, and then incubated overnight at 4 °C with primary antibodies (Table 2). Following three washes in PBS-T, blots were incubated in appropriate secondary antibodies, for 2 h at room temperature. Membranes were washed and developed for 2 min with Clarity™ Western ECL Substrate (Bio-Rad, CA, United States). Imaging was performed using a ChemiDoc™ MP Imaging System with Image Lab™ software (Bio-Rad, CA, United States).

**Table 2:** List of antibodies used for western blot

Antibody	Dilution	Company	Catalogue number
Rabbit Anti-Caspase-1	1:1000	Cell Signaling	E2Z1C
Mouse Anti-Caspase-1	1:1000	Abcam, Cambridge, UK	ab138483
Rabbit anti GSDMD	1:1000	Abcam, Cambridge, UK	EPR19828
Rabbit anti GAPDH	1:3000	Sigma Aldrich, MO, USA	G9545
Goat anti Rabbit Horseradish peroxidase (*HRP)* conjugate	1:1000	Bio-Rad, CA, USA	170–6515
Goat anti mouse Horseradish peroxidase (*HRP)* conjugate	1:1000	Bio-Rad, CA, USA	170–6516

### IL-1β enzyme-linked immunosorbent assay (ELISA)

IL-1β levels in cell culture supernatant, mouse retinal lysates and EV were determined using an IL-1β ELISA (ELISAkit.com, Scoresby, Australia and Themo Fisher Scientific, MA, United States) according to the manufacturer’s instructions.

### Imaging and statistical analysis

Confluency and morphology of primary and immortalised cell cultures was examined using Zeiss Axiovert 200 Fluorescence/Live cell imagine microscope (Zeiss) at 10× and 40× magnification. Fluorescence in retinal and cell sections was visualised using A1^+^ Confocal Microscope System with NIS-Elements Advanced Research software.

RNA sequencing: Statistical analysis for RNA sequencing was performed as specified above. Graphing was generated using ggplot2 package and Prism V7.0. Unpaired Student’s *t* tests or one-way analysis of variance (ANOVA) were performed using Prism V9.2. Adjusted *p* values < 0.1 were deemed statistically significant. All data were expressed as the mean ± SEM. (*N* = 4–5).

Functional analysis (ERG): All graphing and statistical analysis was performed using Prism 9 (GraphPad Software, CA, USA). Two-way analysis of variance (ANOVA) with Šídák or Bonferroni multiple comparison post-hoc test was used to determine the statistical outcome; *p* value of < 0.05 was considered statistically significant. All data were expressed as the mean ± SEM.

Histology: All graphing and statistical analysis was performed using Prism 9 (GraphPad Software, CA, USA). An unpaired Student *t* test, one-way analysis of variance (ANOVA), or two-way ANOVA with Tukey’s multiple comparison post-test were utilised to determine the statistical outcome; *p* value of < 0.05 was considered statistically significant for all histological analysis. All data were expressed as the mean ± SEM.

In vitro experiments: All graphing and statistical analysis was performed using Prism 9 (GraphPad Software, CA, USA). Unpaired *t* test, Mann–Whitney test with false discovery rate (FDR), or two-way ANOVA with Tukey’s or Šídák multiple comparison post-hoc test was used to determine the statistical outcome. *p* value of < 0.05 was considered statistically significant.

*For *in vivo* experiments, 'N' represents the number of animals in the individual experimental run, graphs depict average of technical replicates from multiple measurements per animal. Gsdmd*^*105N/105N*^ DR and PD experiments were repeated thrice (3 individual experimental runs, *N* = 4–7 each) and *Gsdmd*^*−/−*^ DR and PD experiments were repeated twice (2 individual experimental runs, *N = *4–5 each).

## Results

### Pyroptotic pore forming protein Gasdermin D contributes to retinal degeneration

**Fig. 1:**
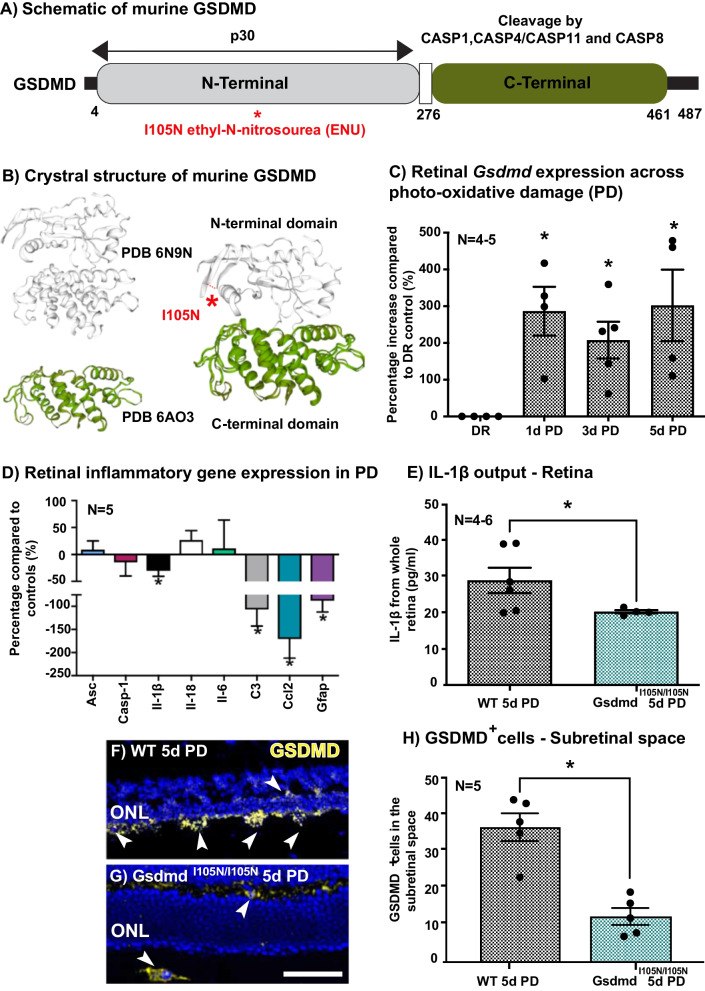
*Gsdmd*^*I105N/I105N*^ mutant characterisation. **A** Schematic of murine GSDMD with I105N substitution site highlighted **B** Crystal structure of N-terminal and C-terminal domain of GSDMD created as superimposition of Protein Data Bank (PDB) 6N9N and PDB 6AO3 X-ray structures using SWISS-MODEL. **C** Retinal expression of *Gsdmd* at 1, 3 and 5 days (5d) PD compared to DR controls as measured by qRT-PCR (p < 0.05, *N = *4–5, ordinary one-way ANOVA with Tukey’s multiple comparison test). **D** Expression of inflammatory genes in the retina, as measured by qRT-PCR, in WT and *Gsdmd*^*I105N/I105N*^ mice following PD. The expression of *IL-1β, C3, Ccl2* and *Gfap* were significantly downregulated in *Gsdmd*^*I105N/I105N*^ mice compared to WT controls (p < 0.05, *N = *5). **E** Total IL-1β release from WT and *Gsdmd*^*I105N/I105N*^ whole retina post-5-day PD as measured by ELISA (p < 0.05, *N = *4–6). **F**, **G** Representative images of GSDMD IHC of WT and *Gsdmd*^*I105N/I105N*^ retina post-5day PD (Scale bar = 50 µm) showing **H** reduced GSDMD^+^ cells in subretinal space of *Gsdmd*^*I105N/I105N*^ mice in comparison with WT post-PD (p < 0.05, *N = *5)

In this work, we investigated the potential role of GSDMD in photo-oxidative damage (PD)-induced retinal degeneration using an ENU-derived *Gsdmd* mutant strain-*Gsdmd*^*I105N/I105N*^ carrying an Ile to Asn amino acid substitution at position 105 (Fig. 1A, B), impairing the pore forming capacity of N-terminal GSDMD. Retinas from WT and *Gsdmd*^*I105N/I105N*^ mice expressed comparable amounts of GSDMD in the dim-reared (DR) condition, suggesting I105N mutation impairs function rather than protein expression (Additional file 1: Fig. S1A).

To explore the role of *Gsdmd* in retinal degeneration, gene expression levels of *Gsdmd* across 1, 3 and 5 days of PD were measured using qRT-PCR (Fig. 1C). Results demonstrated a significant upregulation of *Gsdmd* in retinal lysates at 1, 3, 5 days of PD, peaking in expression at 1 and 5 days of damage (Fig. 1C, p < 0.05). *Gsdmd*^*I105N/I105N*^ mice were further found to have significantly reduced gene expression of inflammatory cytokines/chemokines known to play a role in AMD *Il-1β, C3, Ccl2* and *Gfap* compared to WT controls following 5 days of PD (Fig. 1D, p < 0.05). Upstream inflammasome genes *Asc* and *Casp-1* were unchanged. Furthermore, we saw significantly reduced levels of total IL-1β protein in the retina, as measured by ELISA (Fig. 1E, p < 0.05).

In addition, GSDMD protein expression was visualised in WT and *Gsdmd*^*I105N/I105N*^ mice retinas in both DR conditions (Additional file 1: Fig. S1B) and following 5 days of PD (Fig. 1F, G). Following PD, *Gsdmd*^*I105N/I105N*^ mice were found to have fewer GSDMD^+^ cells in the subretinal space when compared to WT controls. Furthermore, GSDMD^+^ cells in the subretinal space were also IBA-1^+^, a marker of microglia and macrophages, (Additional file 1: Fig. S1C), indicating that GSDMD is primarily expressed in the subretinal macrophages during degeneration.

Taken together these results support a role for GSDMD in the release of pro-inflammatory cytokine IL-1β from retinal microglia/macrophages, which may mediate retinal inflammation in response to PD.

### Impairing Gasdermin D function post-translationally is protective against photo-oxidative damage-induced retinal degeneration

**Fig. 2:**
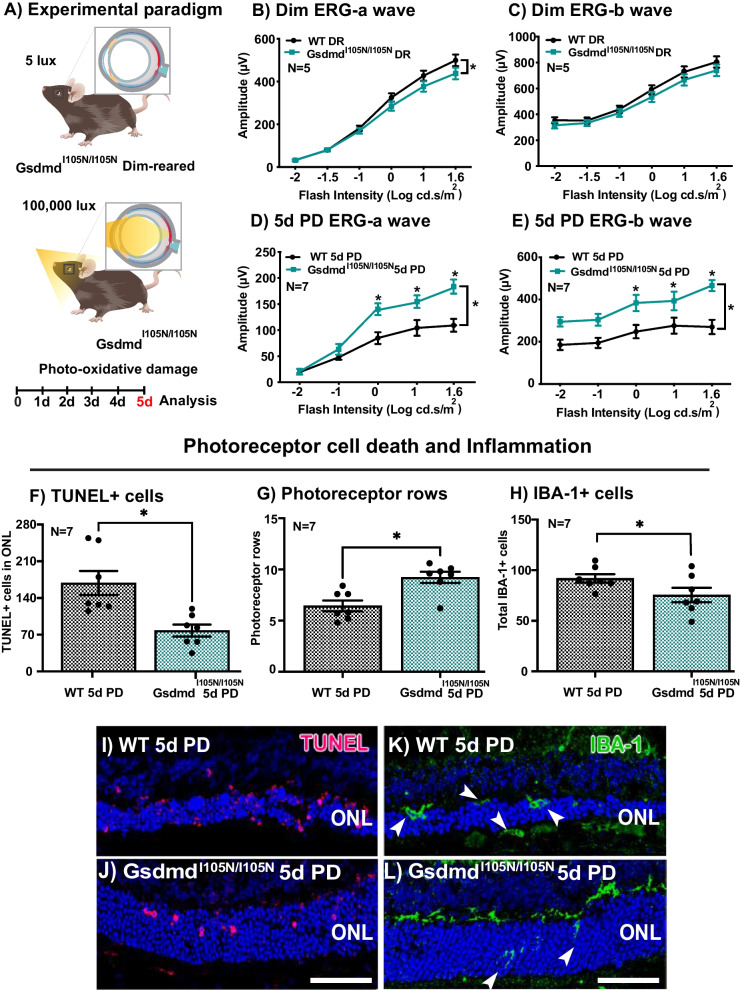
*Gsdmd*^*I105N/I105N*^ mice have improved retinal function, reduced cell death and immune cell recruitment compared to WT controls when subject to 5 days of photo-oxidative damage. **A** Experimental paradigm showcasing mice under DR and PD conditions. Retinal ERG function is significantly higher for WT mice in comparison with *Gsdmd*^*I105N/I105N*^ in DR condition as showcased by **B** a-wave (p < 0.05, *N = *5) with no significant change in **C** b-wave responses (p > 0.05, *N = *5). However, when subject to 5d PD, *Gsdmd*^*I105N/I105N*^ mice have significant protection against degeneration compared to WT as indicated in the retinal ERG trace **D** a-wave (p < 0.05, *N = *7) and **E** b-wave (p < 0.05, *N = *7). *Gsdmd*^*I105N/I105N*^ mice also show reduced photoreceptor cell death as indicated by **F** TUNEL + counts (p < 0.05, *N = *7), **G** Improved retention of photoreceptor rows (p < 0.05, *N = *7) and **H** reduction in recruitment of IBA-1 + cells in response to damage (p < 0.05, *N = *7) **I**, **J** Representative image of TUNEL^+^ assay and **K**, **L** IBA-1^+^ IHC. Scale bar = 50 µm

To further elucidate the contribution of GSDMD in the progression of retinal degeneration, we assessed the retinal function and histology of *Gsdmd*^*I105N/I105N*^ mice in both DR conditions and following 5 days of PD, compared to WT controls. ERG demonstrated that DR *Gsdmd*^*I105N/I105N*^ mice had reduced retinal function for a-wave response (Fig. 2B, p < 0.05) but no change in b-wave response (Fig. 2C, p > 0.05) compared to WT controls. However, following 5 days of PD, *Gsdmd*^*I105N/I105N*^ mice had significantly higher a-wave and b-wave responses than WT mice (Fig. 2D, E, p < 0.05). Individual amplitudes of a-wave and b-wave responses at maximum flash intensity 1.6 log cd s m^−2^for both DR and PD mice has been provided in Additional file 2: Fig. S2. The preserved retinal function in *Gsdmd*^*I105N/I105N*^ mice was reflected by both significantly decreased numbers of TUNEL^+^ cells in the ONL (Fig. 2F, p < 0.05), and increased photoreceptor row counts (Fig. 2G, p < 0.05). Representative confocal images show increased TUNEL^+^ cells and ONL thinning in WT mice (Fig. 2I), compared to *Gsdmd*^*I105N/I105N*^ mice (Fig. 2J).

Retinal inflammation was also measured in WT and *Gsdmd*^*I105N/I105N*^ mice following 5 days of PD, *Gsdmd*^*I105N/I105N*^ mice were found to have significantly reduced IBA-1^+^ cells in the outer retina compared to controls (Fig. 2H, p < 0.05). Representative confocal images show the reduced presence of IBA-1^+^ cells in the retina of *Gsdmd*^*I105N/I105N*^ mice following 5 days of PD compared to WT controls (Fig. 2K, L).

Considering GSDMD expression was shown to co-localise with subretinal IBA-1^+^ cells, these results further support a potential role for pyroptotic pore protein GSDMD in mediating inflammatory cascades, with inhibition shown to protect the retina against degeneration.

### Impairing Gasdermin D protein translation does not provide protection against retinal inflammation

**Fig. 3:**
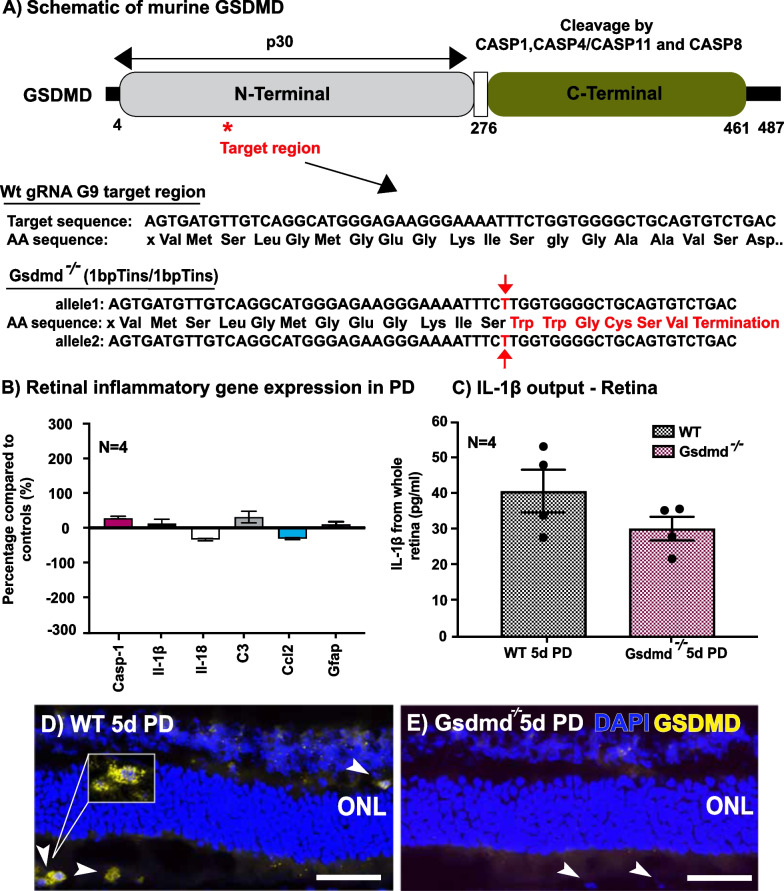
*Gsdmd*^*−/−*^ CRISPR knockout characterisation. **A** Schematic of murine GSDMD with CRISPR G9 target region highlighted, and corresponding amino acid sequence decoded. **B** Retinal gene expression panel of inflammatory genes, as measured by qRT-PCR, in WT and *Gsdmd*^*−/−*^ mice following 5d PD (p > 0.05, *N = *4). **C** IL-1β released from WT and *Gsdmd*^*−/−*^ retina post-5-day PD as measured by ELISA (p > 0.05, *N = *4). **D**, **E** Representative image of GSDMD immunohistochemistry in WT and *Gsdmd*^*−/−*^ retinas post-5day PD. Scale bar = 50 μm

Given the preserved function and increased photoreceptor survivability in *Gsdmd*^*I105N/I105N*^ mice, a possible mechanism of GSDMD-mediated protection was investigated via the use of a CRISPR-mediated knockout strain, which has been referred to as *Gsdmd*^*−/−*^ in this study (Fig. 3A). Frameshift in the *Gsdmd* ORF truncates the coding sequence as described in Kayagaki et al. 2015, impairing not just function but also synthesis of GSDMD in the retina as shown in the immunoblot of DR mice (Additional file 3: Fig. S3).

Unlike *Gsdmd*^*I105N/I105N*^ mice, *Gsdmd*^*−/−*^ mice when exposed to 5 days of PD, displayed comparable gene expression levels of *Il-1β, Il-18, C3, Ccl2* and *Gfap* to WT controls (Fig. 3B, p > 0.05). No significant change was seen in levels of total IL-1β protein in the retina, as measured by ELISA (Fig. 3C, p > 0.05). In addition, GSDMD protein expression was visualised in WT and *Gsdmd*^*−/−*^ mice retinas following 5d PD (Fig. 3D). However, no GSDMD expression was observed in plexiform layers or subretinal macrophage cells in *Gsdmd*^*−/−*^ mice likely due to the CRISPR knockout truncating GSDMD production.

**Fig. 4:**
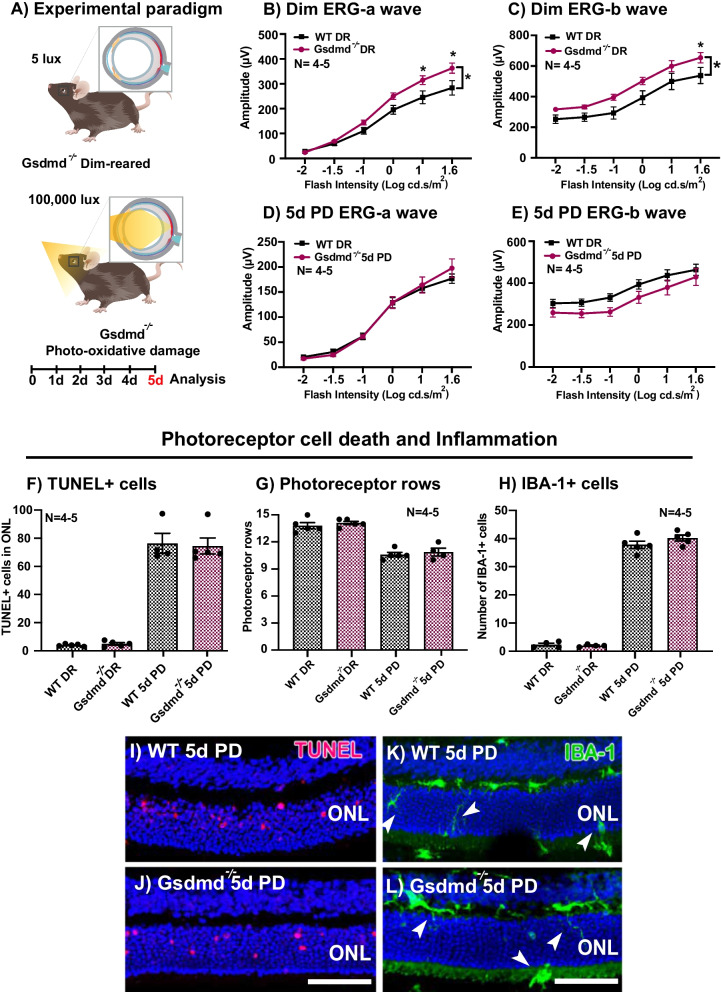
*Gsdmd*^*−/−*^ mice have no significant change in retinal function, cell death and immune cell recruitment compared to WT controls when subject to 5-day photo-oxidative damage. **A** Experimental paradigm showcasing mice under DR and PD conditions. Retinal ERG function is significantly higher for WT mice in comparison with *Gsdmd*^***−/−***^ in DR condition as showcased by **B** a-wave and **C** b-wave responses (p < 0.05, *N = *4–5). However, when subject to PD, both WT and *Gsdmd*^*−/−*^ mice show comparable levels of degeneration as indicated in the retinal ERG traces for **D** a-wave and **E** b-wave (p > 0.05, *N = *4–5). *Gsdmd*^*−/−*^ mice also show comparable levels of photoreceptor cell death as indicated by **F** TUNEL^+^ counts (p > 0.05, *N = *4–5), **G** photoreceptor rows (p > 0.05, n = 4–5) and **H** recruitment of IBA-1^+^ cells in DR condition and in response to damage (p > 0.05, n = 4–5) **I**, **J** Representative image of TUNEL assay and **K**, **L** IBA-1^+^ immunohistochemistry. Scale bar = 50 µm

To identify if mice with truncated GSDMD protein may confer equal or higher levels of overall retinal protection to WT in comparison to  *Gsdmd*^*I105N/I105N*^, retinal function and histology of *Gsdmd*^*−/*−^ mice and WT mice were assessed in both DR condition and following 5 days of PD (Fig. 4A). ERG revealed that DR *Gsdmd*^*−/−*^ mice had slightly increased retinal function for both a-wave and b-wave responses compared to WT controls (Fig. 4B, C, p < 0.05). However, following 5 days of PD, *Gsdmd*^*−/−*^ mice had comparable a-wave and b-wave responses to WT mice (Fig. 4D, E, p > 0.05), indicating no level of functional protection in *Gsdmd*^*−/−*^ mice against retinal degeneration. Individual amplitudes of a-wave and b-wave responses at maximum flash intensity 1.6 log cd s m^−2^for both DR and PD mice has been provided in Additional file 4: Fig. S4.

In addition, there was no significant difference in total TUNEL^+^ cell counts in the ONL (Fig. 4F, p > 0.05), or number of photoreceptor rows (Fig. 4G, p > 0.05), between these groups in both DR conditions and post-5-day PD. Retinal inflammation was measured using IBA-1 labelling, and showed no significant change in number of IBA-1^+^ cells in the outer retina between WT and *Gsdmd*^*−/−*^ mice in both DR and 5d PD conditions (Fig. 4H, p > 0.05). Representative confocal images depict the results of TUNEL^+^ assay (Fig. 4I, J), and IBA-1 IHC (Fig. 4K, L).

Taken together these results indicate that impairing production of GSDMD is not protective against PD-induced retinal degeneration. However, impairing GSDMD function post-production, might pose a therapeutic opportunity. We reason that this could be due to a compensatory mechanism in play in the absence of full-length GSDMD protein in the retina during degeneration as reported by Wang and colleagues [47].

### Gasdermin D mediates NF-κB inflammatory cascade contributing to retinal degeneration

**Fig. 5:**
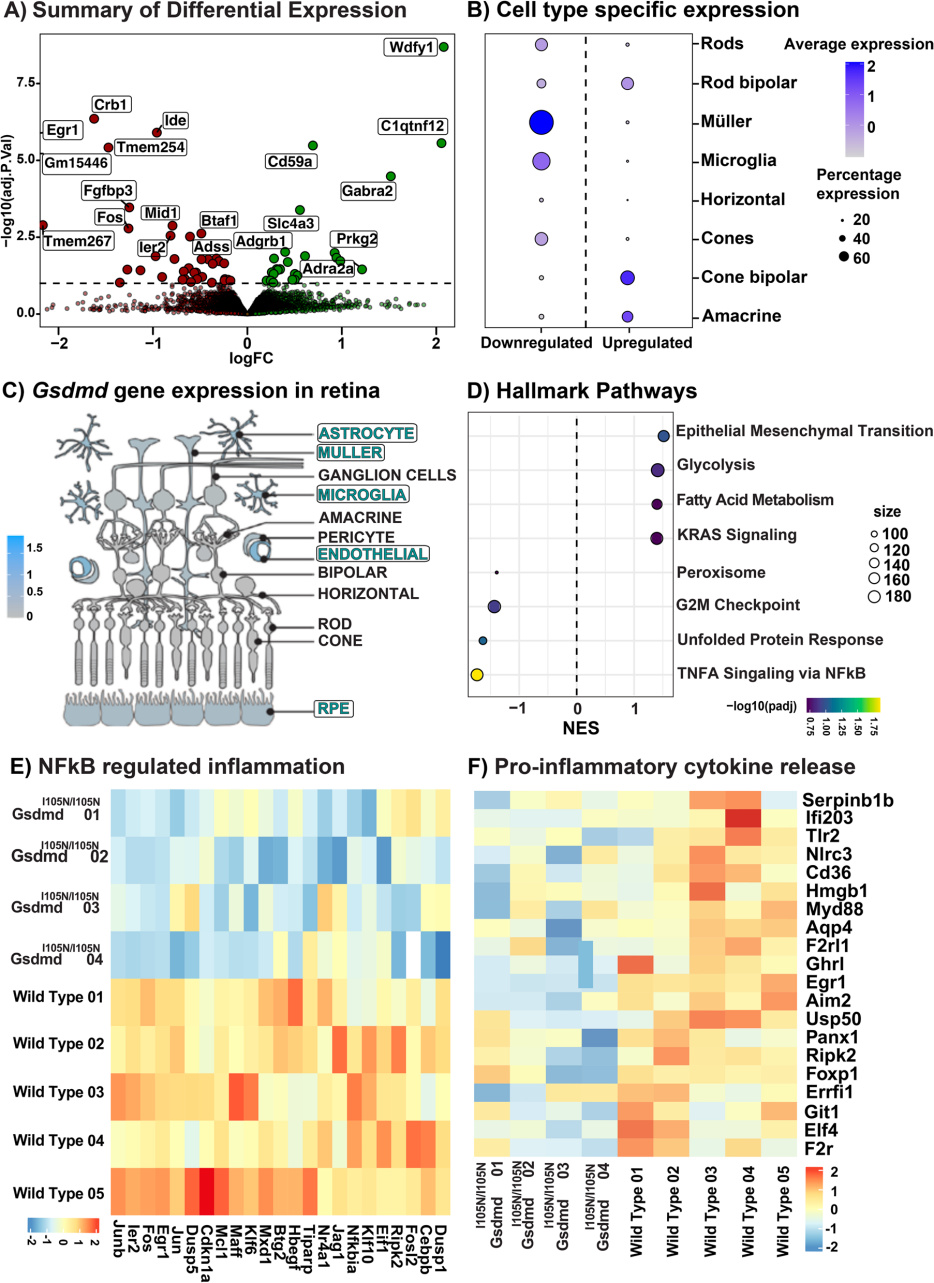
WT and *Gsdmd*^*I105N/I105N*^ mice retinal RNA profiling, enrichment analysis and pathway analysis. **A** Volcano plot indicating top 20 differentially expressed genes in *Gsdmd*^*I105N/I105N*^ mice retinas compared to WT mice retina post-PD (adj.p.value < 0.1, *N = *4–5). **B** Retinal cell types enriched in genes differentially expressed between *Gsdmd*^*I105N/I105N*^ and WT retinas post-PD (logFC < or > 1). **C** Illustration showcasing human and mouse cell types that express *Gsdmd* in the retina. **D** Biological processes that are positively and negatively enriched in *Gsdmd*^*I105N/I105N*^ compared to WT retinas post-PD (adj.p.value, *N = *4 -5). Heatmaps showcasing downregulation in genes associated with **E** NF-κB regulated inflammation and **F** release of pro-inflammatory cytokines in *Gsdmd*^*I105N/I105N*^ mice retinas in comparison with WT mice retinas post-PD

Given the observed protection in *Gsdmd*^*I105N/I105N*^ mice against retinal degeneration, retinal lysates from *Gsdmd*^*I105N/I105N*^ and WT mice underwent RNA sequencing to identify transcriptomic changes contributing to the observed protection (Additional file 5: Fig. S5A). Following normalization (Additional file 5: Fig. S5B, C), principal component analysis (PCA) identified distinct clustering between groups as visualised in the 3D plot containing PC1, PC2, and PC3 (Additional file 5: Fig. S5D), showing some within-group variations. Gene expression analysis showed 63 genes were differentially expressed (adj.p.val < 0.1) between *Gsdmd*^*I105N/I105N*^ and WT mice (Additional file 5: Fig. S5E) following PD, of which the top 20 are visualised in the volcano plot (Fig. 5A, adj.p.val < 0.1).

To determine the potential cellular locations of the differentially expressed genes, their expression in neural retina was investigated using publicly available single cell RNA-seq data set from healthy C57BL/6J mice [44]. Upregulated genes (logFC > 1) were predominantly located in retinal interneurons (amacrine and bipolar cells) (Fig. 5B), whereas downregulated genes (logFC < -1) were predominantly identified in the Müller cells and microglia cluster (Fig. 5B), both of which are known to participate in the inflammatory response in the retina. Low levels of *Gsdmd* expression were also found to co-localise in retinal cell types that were identified as cellular locations of the downregulated genes (Fig. 5C, illustration obtained using Spectacle with reference human and mouse retina single cell RNAseq data sets [48–52]).

Furthermore, to identify the biological processes associated with the differentially expressed genes, pathway analysis was performed using publicly available MSigDB gene sets[53]. Results showed decreased enrichment in peroxisome response, unfolded protein response, and TNFα signalling via NF-κB, indicating overall reduction in inflammation and oxidative stress in *Gsdmd*^*I105N/I105N*^ mice (Fig. 5D, Additional file 6: Fig. S6). Enrichment in epithelial mesenchymal transition (EMT) genes was a result of within-group variations in WT and *Gsdmd*^*I105N/I105N*^ samples and showed no distinct clustering between groups (Additional file 6: Fig. S6).

Decreased enrichment in TNFα signalling via NF-κB corresponded to reduced expression of genes in NF-κB pathway (Fig. 5E), resulting in downregulation of genes associated with the release of pro-inflammatory cytokines, including *Serping1*, *Tlr2*, *Panx1* and *Foxp1*—known drivers of inflammation during AMD (Fig. 5F). These results support the functional and histological protection showcased by *Gsdmd*^*I105N/I105N*^ mice in response to PD.

### Gasdermin D-dependent secretion of IL-1β may contribute to retinal degenerations via release in extracellular vesicles (EVs)

**Fig. 6:**
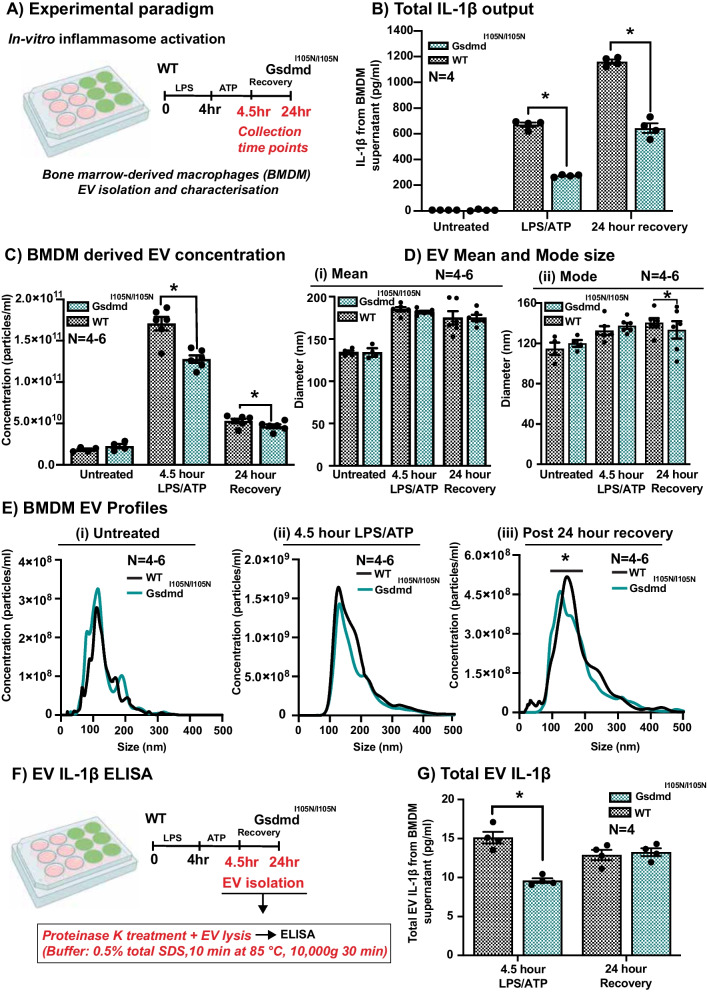
WT and *Gsdmd*^*I105N/I105N*^ BMDM EV profiling. **A** Experimental paradigm showcasing *in-vitro* inflammasome activation of BMDM. **B** IL-1β release from WT and *Gsdmd*^*I105N/I105N*^ BMDM post-LPS/ATP stimulation, as measured by ELISA (p < 0.05, *N = *4). **C** EV concentration measured using nanoparticle tracking analysis (NTA) was significantly reduced in the supernatant of BMDM from *Gsdmd*^*I105N/I105N*^ mice compared to WT controls, following stimulation with LPS/ATP (p < 0.05, *N = *4–6). This effect was also seen following 24 h of recovery (p < 0.05, *N = *4–6). **D** EV mean and mode size (in nm) measured using NTA showing no change in mean size (p > 0.05, *N = *4–6) but shift in mode size (p < 0.05, *N = *4–6). This shift in size is reflected in **E** BMDM EV profiles showing size distribution data obtained from NTA. There is no change in EV profiles in untreated condition (i) or 4.5-h post-LPS/ATP stimulation (ii), however, there is a shift in profile at 24-h recovery timepoint (iii). **F, G** EV IL-1β release from WT and *Gsdmd*^*I105N/I105N*^ BMDM post-LPS/ATP stimulation, as measured by ELISA (p < 0.05, *N = *4)

**Fig. 7:**
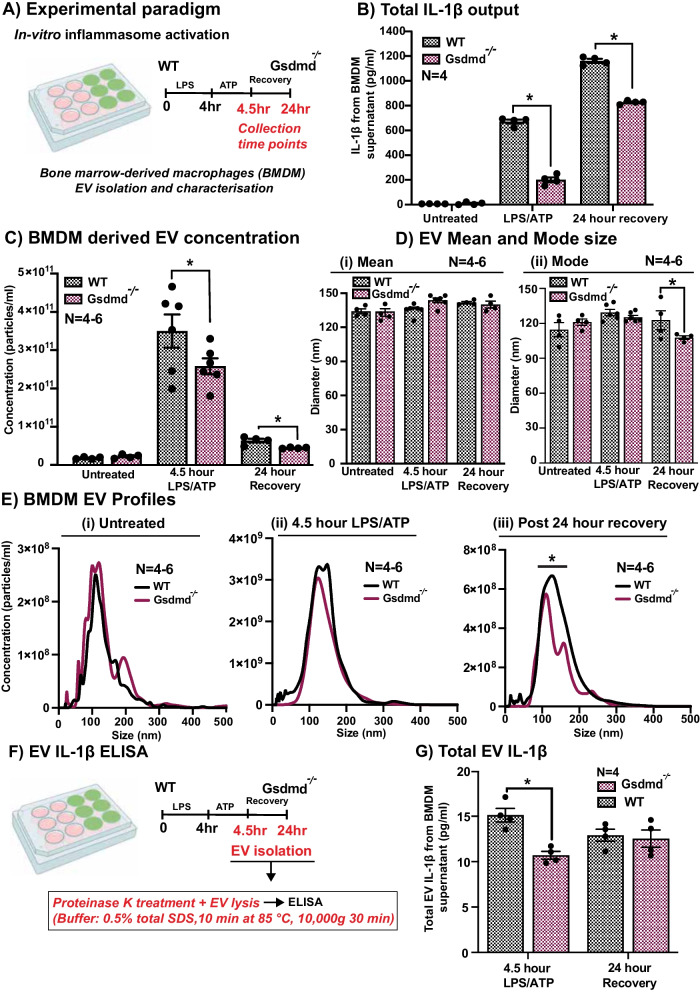
WT and *Gsdmd*^*−/−*^ BMDM EV profiling. **A** Experimental paradigm showcasing *in-vitro* inflammasome activation of BMDM. **B** IL-1β release from WT and *Gsdmd*^*−/−*^ BMDM post-LPS/ATP stimulation, as measured by ELISA (p < 0.05, *N = *4). **C** EV concentration measured using nanoparticle tracking analysis (NTA) was significantly reduced in the supernatant of BMDM from *Gsdmd*^*−/−*^ mice compared to WT controls, following stimulation with LPS/ATP (p < 0.05, *N = *4–6). This effect was also seen following 24 h of recovery (p < 0.05, *N = *4–6). **D** EV mean and mode size (in nm) measured using NTA showing no change in mean size (p > 0.05, *N = *4–6) but shift in mode size (p < 0.05, *N = *4–6) at 24-h recovery timepoint. This shift in size is reflected in **E** BMDM EV profiles showing size distribution data obtained from NTA. There is no change in EV profiles in untreated condition (i) or 4.5-h post-LPS/ATP stimulation (ii), however, there is a shift in profile at 24-h recovery timepoint (iii). **F**, **G** EV IL-1β release from WT and *Gsdmd*^*−/−*^ BMDM post-LPS/ATP stimulation, as measured by ELISA (p < 0.05, *N = *4)

As IL-1β has been strongly implicated in AMD pathogenesis and is known to be released following inflammasome activation, we investigated GSDMD-dependent IL-1β release as the predominant secreted cytokine from GSDMD expressing microglia/macrophages in the retina during degeneration. We investigated the mechanism of GSDMD-dependent pro-inflammatory cytokine secretion using BMDM isolated from WT, *Gsdmd*^*I105N/I105N*^ (Fig. 6A) and *Gsdmd*^*−/−*^ mice (Fig. 7A). Following LPS/ATP stimulation, *Gsdmd*^*I105N/I105N*^ and *Gsdmd*^*−/−*^ BMDM released significantly reduced levels of IL-1β compared to WT controls, as measured by ELISA (Figs. 6B, 7B, p < 0.05). This effect was further seen after 24 h of recovery following LPS/ATP stimulation (Figs. 6B, and7B, p < 0.05).

As recent evidence suggests that GSDMD may play a role in the release of EVs during inflammation, we also investigated GSDMD-dependent EV release using BMDM isolated from WT, *Gsdmd*^*I105N/I105N*^ and *Gsdmd*^*−/−*^ mice. Results showed that under control conditions there was no difference in the concentration of secreted EVs from WT, *Gsdmd*^*I105N/I105N*^ or *Gsdmd*^*−/−*^ BMDM (Figs. 6C, 7C, p > 0.05). However, following LPS/ATP stimulation there were significantly reduced levels of EVs secreted from both *Gsdmd*^*I105N/I105N*^ and *Gsdmd*^*−/−*^ BMDM compared to WT controls (Figs. 6C, 7C, p < 0.05). This effect was further seen after 24 h of recovery following LPS/ATP stimulation (Figs. 6C, 7C, p < 0.05). There was no significant change in mean size of EVs between the two groups (Figs. 6Di, 7D i, p > 0.05). However, there was a shift in mode size of EVs seen after 24 h of recovery following LPS/ATP stimulation (Figs. 6Dii, 7Dii, *p* < 0.05), reflected in the size-distribution graphs (Figs. 6E, 7E, p < 0.05).

Finally, to elucidate if GSDMD-dependant EV release may play a role in IL-1β secretion from BMDM, total EV IL-1β was measured from *Gsdmd*^*I105N/I105N*^ and *Gsdmd*^*−/−*^ supernatant and compared to WT controls following LPS/ATP stimulation (Figs. 6F, 7F). As total EVs isolated from supernatants of *Gsdmd*^*I105N/I105N*^ BMDM had reduced levels of IL-1β in comparison with total EVs from WT controls (Figs. 6G, 7G, p < 0.05), we attribute this to reduced levels of EVs secretion by *Gsdmd*^*I105N/I105N*^ and *Gsdmd*^*−/−*^ BMDM upon stimulation.

This result suggests that GSDMD may mediate inflammation in distant cells by release of pro-inflammatory cytokines, including IL-1β via EVs, especially from phagocyte populations during degeneration. However, as only a small percentage of total IL-1β is encapsulated in EV, there must exist other parallel mechanisms of GSDMD-mediated IL-1β secretion.

### GSDMD inhibition using RNAi may ameliorate inflammation

**Fig. 8:**
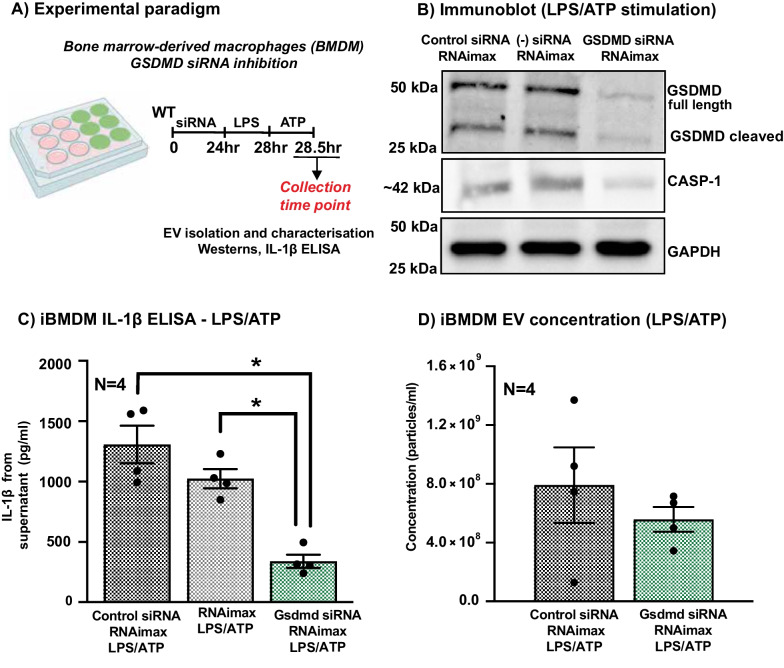
GSDMD inhibition reduces release of pro-inflammatory cytokine IL-1β. **A** Experimental paradigm showcasing *in-vitro* siRNA inhibition followed by inflammasome activation of iBMDM. **B** Representative, western blots showing bands of 42 kDa for CASP-1, ~ 50 kDa for full-length GSDMD and ~ 30 kDa for cleaved GSDMD and 37 kDa for GAPDH reference protein, in iBMDM lysates. **C** Total IL-1β release from iBMDM post-LPS/ATP stimulation, as measured by ELISA (p < 0.05, *N = *4). **D** EV concentration measured using NTA in the supernatant of iBMDM with GSDMD-specific siRNA compared to non-specific siRNA control, showing a reduction in EV number but no significant change (p > 0.05, *N = *4)

Finally, to test the potential of therapeutically targeting GSDMD to ameliorate inflammation, a GSDMD-specific siRNA was encapsulated in Lipofectamine RNAimax to inhibit GSDMD function in immortalised BMDM (iBMDM) and compared to a non-specific siRNA control (Fig. 8A). Following inflammasome stimulation, GSDMD protein level was reduced in the iBMDM cell lysate with GSDMD-specific siRNA (25 pmol) compared to those with non-specific negative siRNA control or just Lipofectamine RNAimax (Fig. 8B). iBMDM with GSDMD-specific siRNA also released reduced amounts of IL-1β in cell-free supernatant, compared to both non-specific negative siRNA control and Lipofectamine RNAimax (Fig. 8C, p < 0.05). This effect was comparable to the reduced amount of IL-1β in cell-free supernatant of *Gsdmd*^*I105N/I105N*^ and *Gsdmd*^*−/−*^ BMDM compared to WT controls.

To verify if RNAi-mediated inhibition of GSDMD in iBMDM might affect EV release as seen in GSDMD-deficient primary BMDM, total concentration of EV in cell-free supernatant was measured using NTA. Although iBMDM with GSDMD-specific siRNA showed a reduction in EV release post-LPS/ATP stimulation, in comparison with non-specific siRNA control, this difference was not significant (Fig. 8D, p > 0.05).

Overall, we demonstrate increased retinal expression of GSDMD in degeneration, and show that inhibition of GSDMD function *in-vivo* animal models of retinal degeneration and *in-vitro* reduces release of pro-inflammatory cytokine IL-1β, providing overall protection. We also suggest that increased EV production in phagocyte populations mediated by GSDMD may contribute to the pathogenesis of retinal degenerations potentially via the transport of IL-1β and other pro-inflammatory cytokines to propagate inflammation, this has been further investigated in Additional file 7: Fig. S7.

## Discussion

This work has demonstrated an important function of the pyroptotic pore-forming protein GSDMD in mediating inflammatory responses underlying the progression of retinal degenerations. We showed that GSDMD expression is upregulated in the retina following degeneration and localised primarily to subretinal macrophages. Mice deficient in GSDMD function were found to have significantly preserved retinal function, increased photoreceptor survivability, and decreased inflammation compared to controls following photo-oxidative damage-induced retinal degeneration. Furthermore, using RNAseq analysis of whole retinas post-PD, we showed that *Gsdmd*^*I105N/I105N*^ mice had downregulated expression of NF-κB-mediated signalling pathways, a well-known pro-inflammatory cascade expressed in endothelial cells, glia, and astrocytes in the retina [54], and is described as a biomarker of inflammation. In line with recent evidence, activated macrophages from GSDMD-deficient mice were found to release significantly fewer EV compared to WT controls and had significantly reduced IL-1β levels in total cell-free supernatant and in the EV. Taken together, we attribute the downregulation in inflammatory pathways in the whole retina to reduced GSDMD-dependent release of potent pro-inflammatory cytokine IL-1β, likely mediated via EV, which may prevent the propagation of inflammation in distal recipient cells. We also present findings demonstrating the efficacy of targeting this downstream inflammasome effector using siRNA and a pharmacological agent *in-vitro* to partially block IL-1β loaded EVs, these results offer potential avenues for advancing future therapeutic strategies, including *in-vivo* testing.

### Gasdermin D in propagation of retinal degenerations

GSDMD is regarded as the pyroptosis executioner of downstream inflammasome signalling, forming an essential part of the innate immune system [11]. Following cleavage by inflammasome effector CASP-1, GSDMD forms pores on the plasma membrane that subsequently results in cell lysis and the expulsion of pro-inflammatory mediators [6, 32, 55]. Among these mediators, the secretion of IL-1β is particularly well-characterized in the retina and has been shown to have strong associations with the pathogenesis of retinal degenerative diseases, including AMD in cell culture, animal models, and human studies, [8, 18, 34]. Given the correlation between GSDMD-mediated pore formation and IL-1β release, it appears likely that GSDMD would also be involved in disease pathogenesis and represents a potential therapeutic target. Results from this work strongly support a role for GSDMD in the pathogenesis of retinal degeneration, with GSDMD-mutant mice demonstrating improved retinal function, reduced levels of cell death and inflammation. Furthermore, the number of GSDMD expressing microglia/macrophage in the subretinal space was significantly reduced in GSDMD-mutant mice, along with reduced levels of IL-1β.

Other studies support this role for GSDMD deficiency in protecting against retinal degeneration, with Sonny et al., (2023) demonstrating that GSDMD deficiency caused an overall reduction in retinal thinning and a decrease in total number of activated microglia/macrophages compared to WT littermates, in a hyperoxia induced model of retinopathy [56]. Furthermore, findings from their transcriptome analyses suggest that GSDMD-deficient animals were relatively resistant to hyperoxic damage as they were able to better regulate the inflammatory response through cAMP, TNF-α, IL-17, and NF-κB signalling [56]. This is in line with our RNAseq analysis for *Gsdmd*^*I105N/I105N*^ mice post-PD, which indicate that GSDMD-deficiency downregulates pro-inflammatory cascade mediated by NF-kB, dampening overall immune response. Significant downregulation of genes identified in this study, such as *Fos, Cebpb and Junb*, all key transcription factors involved in modulating immune cell activation and encoding family of IL-1 pyrogens [57–59], when taken together could contribute to reduced inflammation, cell death and improved retinal protection, along with reduced IL-1β secretion.

The most well-characterised role of GSDMD is in the secretion of pro-inflammatory cytokines, especially IL-1β, following inflammasome activation. Understanding how IL-1β is released and propagated far from the initial damage site is vital in combating retinal inflammation and slowing disease progression. EV-mediated release of IL-1β has been proposed as a potential strategy employed by GSDMD in propagating inflammation [21, 60]; however, there is little understanding on GSDMD-mediated EV release or its role in IL-1β transport in healthy or diseased retinas.

### Gasdermin Dmediated release of IL-1β in extracellular vesicles

In addition to its function as pyroptosis effector protein, GSDMD has most recently been suggested to play a role in the secretion of EVs [14, 21, 23, 61]. EVs are nanoscale particles [62–64] that mediate homeostasis and immune modulation through the transfer of genetic contents from host to recipient cells [65–70]. It is proposed that the formation of pyroptotic pores triggers changes in the transmembrane potential of the cell, resulting in influx of calcium from the extracellular space, which is detected by lysosomal calcium sensory proteins of the Synaptotagmin family [71, 72]. These proteins promote fusion of endosomes and lysosomes with the plasma membrane, resulting in release of vesicles from the damage site and enabling return to homeostasis [14, 26]. This repair mechanism is mediated by ESCRT machinery, a central regulator of EV biogenesis and release [23, 73–75]. Though this machinery has been previously established in certain non-ocular disease models [21, 24], the role of GSDMD-dependent EV secretion in the retina has not been well-investigated.

As GSDMD in the retina was found to primarily localize to subretinal macrophage populations [16, 76] following PD, the GSDMD-dependent secretion of EVs was investigated using primary BMDM derived from both WT- and GSDMD-deficient mice. In this work we demonstrate that GSDMD-deficient mice display significantly reduced EV secretion compared to WT controls. This was shown in cells directly following stimulation and 24 h later with LPS/ATP, a known inflammasome activator. In addition, we show that in GSDMD-deficient mice, total EV IL-1β content from activated macrophages was significantly reduced compared to WT controls. This aligns with studies conducted by Bulek and colleagues (2020) that demonstrated that GSDMD may guide the release of IL-1β-containing EVs in epithelial cells to regulate inflammation and a return to homeostasis [21]. This provides a new mechanism for GSDMD-mediated retinal degeneration, where IL-1β may be packaged and sent to distant cells via EV to propagate inflammation. However, as in our results, only a fraction of total EV release was inhibited using GW4869-based EV inhibition, it could be beneficial to look at the EV depleted supernatant to explore alternate IL-1β release mechanisms that may have been in play in parallel.

Considering the implicated role of EV and IL-1β in immune modulation during retinal degeneration, we propose that impairing GSDMD function in the retina using a GSDMD-specific genetic or pharmacological blocker, or inhibiting GSDMD-dependant EV release in resident immune cells may provide therapeutic protection against retinal degenerations.

### Targeting Gasdermin D-dependent release of IL-1β and EV

Clinical interest in the modulation of various inflammasome components is strongly growing due to their crucial role in innate immunity [6, 7, 32, 77]. A majority of the suggested strategies in the retina target inflammasome components NLRP3 [6, 78, 79], Caspase-1 [5, 80], Caspase-11 [5] or downstream pro-inflammatory cytokines IL-18 [81, 82] and IL-1β [8, 18, 82], with many studies using either siRNA or pharmacological inhibitors, such as MCC950 or CRID3 [5, 32, 79, 83], IFM-632 [84] and IFM-514 [85] to therapeutically target the NLRP3 driven inflammasome cascade. RNAi and pharmacological inhibitors have also been used to target GSDMD-mediated inflammation in non-ocular tissues, these include Necrosulfonamide [86], LDC7559 [87], Disulfiram [88] and Dimethyl fumarate (DMF) [89], C202-2729 [90], all of which have been demonstrated to impair GSDMD function but also have off target effects and have not been approved for use in retinal degenerations.

In this work, we investigated the efficacy of GSDMD-specific siRNA and GW4869 (sphingomyelinase inhibitor that blocks ESCRT-dependent EV release) [91, 92] in inhibiting GSDMD-mediated inflammation in immortalised BMDM. GSDMD-specific siRNA inhibition demonstrated reduced levels of total IL-1β in cell-free supernatant following inflammasome activation, supporting their potential capacity as therapeutic inhibitors for GSDMD-mediated inflammation. Blocking ESCRT-dependant EV release using GW4869 also showed a reduced trend in total EV number released following stimulation, which translated to a significant reduction in total EV IL-1β content, suggesting this may reduce their capacity to induce inflammation in distant cells. However, other studies have suggested that inhibition of sphingomyelinase-mediated ESCRT machinery may exacerbate pyroptosis and IL-1β secretion in immune cells [23, 24, 26, 75], indicating that this machinery may be a double-edged sword, as it plays an essential role in membrane repair to avoid pyroptosis while also regulating packaging of pro-inflammatory cytokines into EV.

This reiterates the need for GSDMD-specific genetic inhibitors and pharmacological inhibitors to be tested in disease-specific, tissue-specific, or cell-specific models of degeneration *in-vivo* due to varying levels of GSDMD-inhibition providing varied levels of protection. For example, in this work, despite primary-BMDM from both *Gsdmd*^*I105N/I105N*^ and *Gsdmd*^*−/−*^ mice secreting reduced total IL-1β and total EV IL-1β content *in-vitro* post-stimulation, CRISPR-mediated *Gsdmd*^*−/−*^ mice did not provide long term protection against retinal inflammation. We reasoned that this could be due to the onset of a genetic compensatory effect in the absence of GSDMD during prolonged neuroinflammation. A study by Wang et al., 2021 observed a similar effect in a mouse model of NLRP3 inflammasomopathy, where in the complete absence of GSDMD during prolonged inflammation, there was unexpected onset of Gasdermin E (GSDME)-dependent cytokine release and pyroptosis, potentially limiting the protection provided by absence of GSDMD [47]. GSDME has also been previously implicated in photoreceptor damage in the mouse retina, which in the absence of GSDMD may further exacerbate photoreceptor cell death, providing no protection in *Gsdmd*^*−/−*^ mice compared to WT as seen in our model of light induced-degeneration [93]. This indicates that repressing GSDME in addition to GSDMD may provide increased protection against photoreceptor atrophy in AMD.

Hence, improving our mechanistic understanding of tissue or cell-specific regulation of individual inflammasome components, such as GSDMD, its related proteins, and the downstream mechanisms is imperative for developing specific and targeted therapeutics for various retinal degeneration.

## Conclusion

Inflammation is a major contributor to the onset and progression of retinal degenerative diseases. As a key executioner of pyroptotic cell death and cytokine release, GSDMD represents an ideal therapeutic target to combat the spread of inflammation and cell death that characterises these diseases. We demonstrate that impairing GSDMD function *in-vivo* preserves retinal function, ameliorates inflammation and cell death, and reduces overall release of pro-inflammatory cytokine IL-1β. We show evidence that GSDMD may propagate inflammation in the retina via the secretion of IL-1β via EVs from microglia/macrophage populations during damage. Developing effective therapeutics to target GSDMD could, therefore, be useful in slowing the progression of retinal degenerations by preventing the release of pro-inflammatory mediators, including IL-1β and dampen overall immune response. This strategy provides therapeutic advantage over targeting individual inflammasome components or single pro-inflammatory cytokines post-release.

### Supplementary Information

**Additional file 1: Figure S1.** Gasdermin D characterisation in DR and PD retina. **A** Western blots showing bands of ~ 50 kDa for full-length GSDMD and 37 kDa for GAPDH reference protein in DR retinal lysates of WT and *Gsdmd*^*I105N/I105N*^ mice. **B** Representative image showing GSDMD expression in WT and *Gsdmd*^*I105N/I105N*^ DR retinal sections. **C** Representative image showing co-localisation of GSDMD and IBA-1 in 5d PD retinal sections from *Gsdmd*^*I105N/I105N*^ and WT mice.

**Additional file 2: Figure S2.*** Gsdmd*^*I105N/I105N*^ retinal ERG function at Flash Intensity 1.6 (Log cd.s/m^2^). **A** Experimental paradigm showcasing mice under DR and PD conditions. Retinal ERG function for *Gsdmd*^*I105N/I105N*^ mice in DR condition. **B** A-wave response (*p* > 0.05). **C** B-wave response (*p* > 0.05) and post-5 days of PD- **D** a-wave response (*p* < 0.05) and **E** b-wave response (*p* < 0.05) in comparison with WT mice at Flash Intensity 1.6 Log cd.s/m^2^ (Right eye and Left eye included).

**Additional file 3: Figure S3.** WT and *Gsdmd*^*-/-*^ mice DR Western Blot*. ***A** Western blots showing bands of ~50 kDa for full-length GSDMD and 37 kDa for GAPDH reference protein in DR retinal lysates of WT and *Gsdmd*^*-/-*^ mice.

**Additional file 4: Figure S4.** *Gsdmd*^*-/-*^ retinal ERG function at Flash Intensity 1.6 (Log cd.s/m^2^). **A** Experimental paradigm showcasing mice under DR and PD conditions. Retinal ERG function for *Gsdmd*^*-/-*^ mice in DR condition. **B** a-wave (*p* < 0.05). **C** b-wave (*p* < 0.05) and post-5 days of PD. **D** a-wave (*p* > 0.05) and **E** b-wave (*p* > 0.05) in comparison with WT mice at Flash Intensity 1.6 Log cd.s/m^2^.

**Additional file 5: Figure S5.** WT and *Gsdmd*^*I105N/I105N*^ mice retinal RNA profiling and enrichment analysis. **A** Experimental paradigm with red retinal labelling indicating lesion site. **B** LogCPM values for raw and normalised counts. **C** Relative Log Expression plots showing effective normalisation. **D** Principal component analysis (PCA) identified distinct clustering between groups as visualised in the 3D plot. **E** Summary of differentially expressed genes with cut-off of adjusted *p* value < 0.1 and < 0.05 (*N* = 4–5).

**Additional file 6: Figure S6.** WT and *Gsdmd*^*I105N/I105N*^ mice retinal RNA profiling and differential expression analysis post-PD Heatmaps showing differential expression profile of genes regulating. **A** Epithelial Mesenchymal Transition (EMT) (adj.p.value <0.1) and **B** Peroxisome signalling (adj.p.value <0.1). **C** Scatter plot and heatmap showing no significant change in expression in EV biogenesis and exosome assembly between WT and *Gsdmd*^*I105N/I105N*^ whole retina post-5-day PD.

**Additional file 7: Figure S7.** iBMDM inhibition using GW4869. **A** Experimental paradigm showcasing *in-vitro* EV inhibition followed by inflammasome activation of iBMDM. **B** Representative, cropped western blots showing bands of 42 kDa for CASP-1, ~50 kDa for full-length GSDMD and ~30 kDa for cleaved GSDMD and 37 kDa for GAPDH reference protein, in iBMDM lysates. **C** EV concentration measured using NTA in the supernatant of iBMDM with GW4869 compared to DMSO control post-LPS/ATP stimulation, showing a reduction in EV number but no significant change (*p* > 0.05, *N* = 4). **D** Total EV IL-1β release from iBMDM post-LPS/ATP stimulation, as measured by ELISA (*p* > 0.05, *N* = 4).

**Additional file 8: Figure S8.** Western Blots. **A** GSDMD Western blot of iBMDM (LPS/ATP) used in Fig. 8B and Additional file 4: Fig. S4B. **B** CASP-1 Western blot of iBMDM (LPS/ATP) used in Fig. 8B. **C** GAPDH loading control optimisation blot of iBMDM (LPS/ATP) used in Fig. 8B. **D** GSDMD and GAPDH western blot of DR retina used in Additional file 1: Fig. S1A.

## Data Availability

Data of this study could be accessed freely under reasonable request. Raw sequencing data associated with this study have been deposited in the NCBI Sequencing Read Archive under BioProject accession PRJNA991248.

## Abbreviations

AEC: Animal Ethics Committee
AMD: Age-related macular degeneration
BMDM: Bone-marrow-derived macrophage
cDNA: Complementary DNA
CO2: Carbon dioxide
DR: Dim-reared
ERG: Electroretinography
EV: Extracellular vesicle
GSDMD: Gasdermin D
HTS: High-throughput sequencing
iBMDM: Immortalised bone-marrow-derived macrophage
IgG: Immunoglobulin G
IL-18: Interleukin-18
IL-1β: Interleukin-1β
INL: Inner nuclear layer
LED: Light-emitting diode
NT and CT: N-terminal and C-terminal
ONL: Outer nuclear layer
PBS: Phosphate-buffered saline
PCA: Principal component analysis
PD: Photo-oxidative damage
qPCR: Quantitative polymerase chain reaction
RNA: Ribonucleic acid
RPE: Retinal pigmented epithelium
RT: Room temperature
siRNA: Small interfering RNA
TMM: Trimmed means of m method
WT: Wild type
