# Supplementary material for: Impairing Gasdermin D-mediated pyroptosis is protective against retinal degeneration
Source: J Neuroinflammation. 2023 Oct 20;20:239. doi: 10.1186/s12974-023-02927-2 (PMC10588253; doi:10.1186/s12974-023-02927-2)
Supplement: Supplementary file 1 — Additional file 1: Figure S1. Gasdermin D characterisation in DR and PD retina. A Western blots showing bands of ~ 50 kDa for full-length GSDMD and 37 kDa for GAPDH reference protein in DR retinal lysates of WT and GsdmdI105N/I105N mice. B Representative image showing GSDMD expression in WT and GsdmdI105N/I105N DR retinal sections. C Representative image showing co-localisation of GSDMD and IBA-1 in 5d PD retinal sections from GsdmdI105N/I105N and WT mice. [file 12974_2023_2927_MOESM1_ESM.docx]

**Supplementary Figure 1**

**
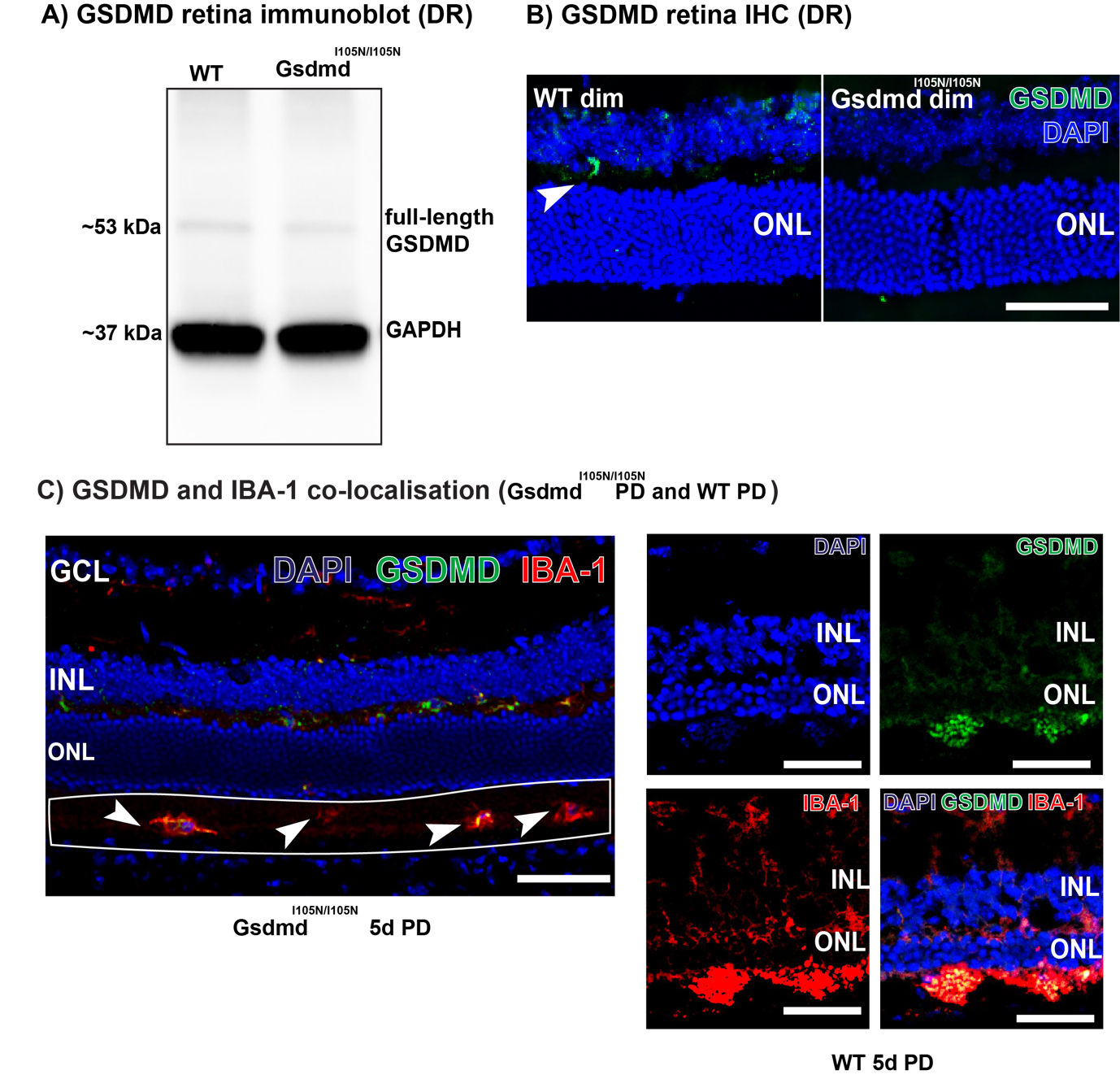
Supplementary Figure 1: Gasdermin D characterisation in DR and PD retina (A)** Western blots showing bands of ~50 kDa for full-length GSDMD and 37 kDa for GAPDH reference protein in DR retinal lysates of WT and *Gsdmd^I105N/I105N^* mice. **(B)** Representative image showing GSDMD expression in WT and *Gsdmd^I105N/I105N^* DR retinal sections. **(C)** Representative image showing co-localisation of GSDMD and IBA-1 in 5d PD retinal sections from *Gsdmd^I105N/I105N^* and WT mice.
