# Supplementary material for: Impairing Gasdermin D-mediated pyroptosis is protective against retinal degeneration
Source: J Neuroinflammation. 2023 Oct 20;20:239. doi: 10.1186/s12974-023-02927-2 (PMC10588253; doi:10.1186/s12974-023-02927-2)
Supplement: Supplementary file 3 — Additional file 3: Figure S3. WT and Gsdmd-/- mice DR Western Blot. A Western blots showing bands of ~50 kDa for full-length GSDMD and 37 kDa for GAPDH reference protein in DR retinal lysates of WT and Gsdmd-/- mice. [file 12974_2023_2927_MOESM3_ESM.docx]

**Supplementary Figure 3**

**
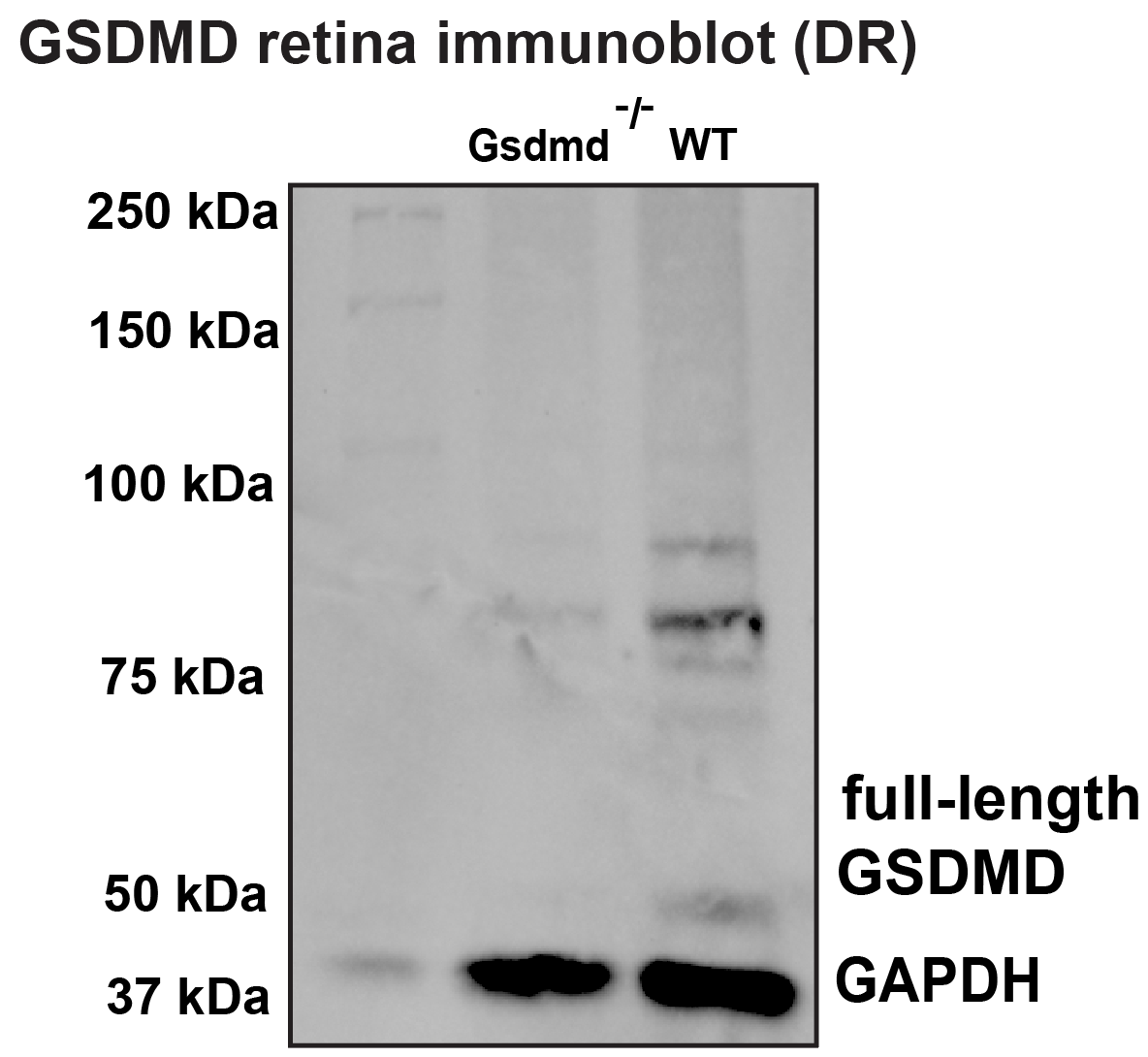
**

**Supplementary Figure 3 :** (A) Western blots showing bands of ~50 kDa for full-length GSDMD and 37 kDa for GAPDH reference protein in DR retinal lysates of WT and *Gsdmd^-/-^* mice.
