# Supplementary material for: Impairing Gasdermin D-mediated pyroptosis is protective against retinal degeneration
Source: J Neuroinflammation. 2023 Oct 20;20:239. doi: 10.1186/s12974-023-02927-2 (PMC10588253; doi:10.1186/s12974-023-02927-2)
Supplement: Supplementary file 4 — Additional file 4: Figure S4. Gsdmd-/- retinal ERG function at Flash Intensity 1.6 (Log cd.s/m2). A Experimental paradigm showcasing mice under DR and PD conditions. Retinal ERG function for Gsdmd-/- mice in DR condition. B a-wave (p < 0.05). C b-wave (p < 0.05) and post-5 days of PD. D a-wave (p > 0.05) and E b-wave (p > 0.05) in comparison with WT mice at Flash Intensity 1.6 Log cd.s/m2. [file 12974_2023_2927_MOESM4_ESM.docx]

**Supplementary Figure 4**

**
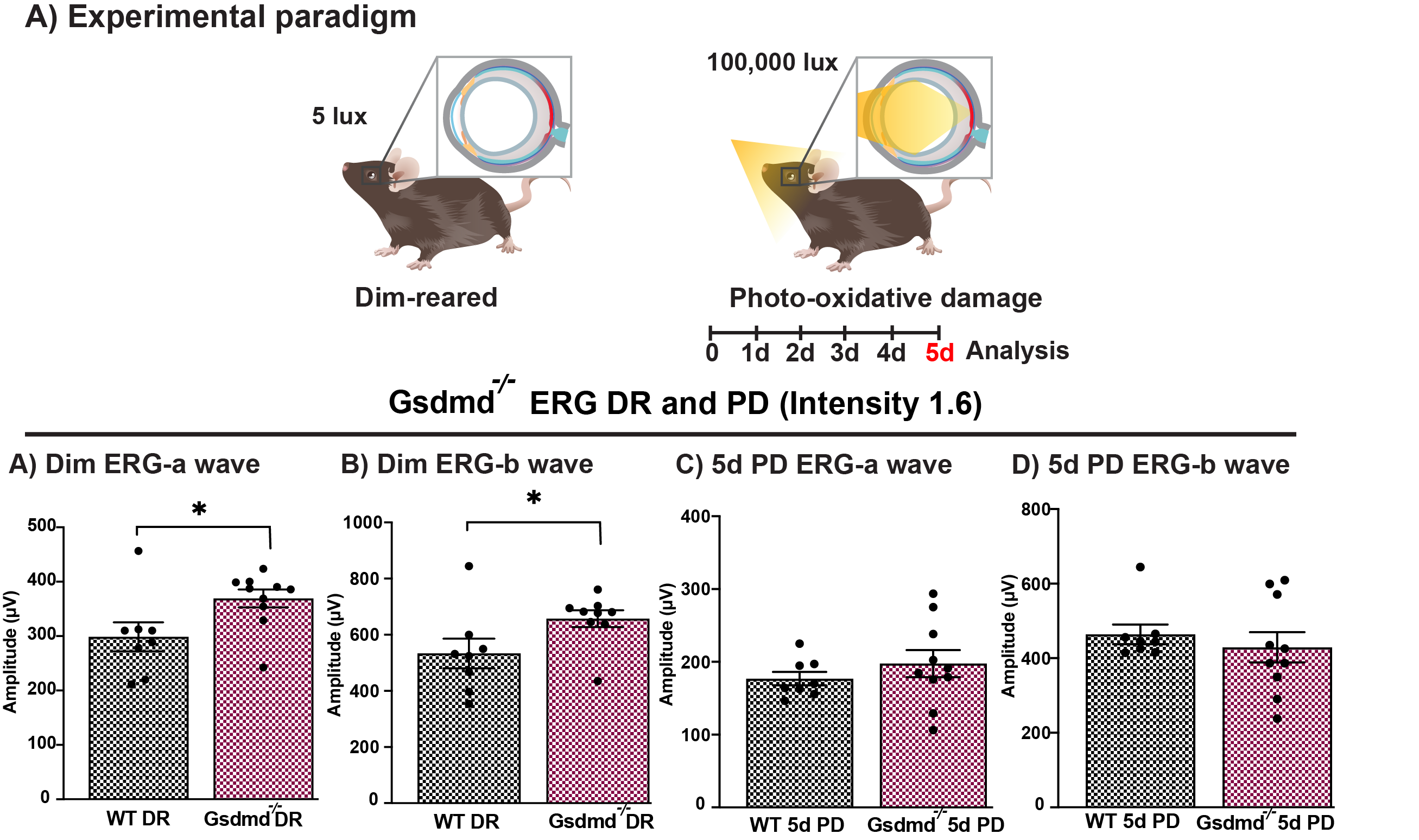
**

**Supplementary Figure 4: *Gsdmd^-/-^* retinal ERG function at Flash Intensity 1.6 (Log cd.s/m^2^). (A)** Experimental paradigm showcasing mice under DR and PD conditions. Retinal ERG function for *Gsdmd^-/-^* mice in DR condition (B) a-wave (p<0.05) (C) b-wave (p<0.05) and post 5 days of PD- (D) a-wave (p>0.05) and (E) b-wave (p>0.05) in comparison to WT mice at Flash Intensity 1.6 Log cd.s/m^2^.
