# Supplementary material for: Impairing Gasdermin D-mediated pyroptosis is protective against retinal degeneration
Source: J Neuroinflammation. 2023 Oct 20;20:239. doi: 10.1186/s12974-023-02927-2 (PMC10588253; doi:10.1186/s12974-023-02927-2)
Supplement: Supplementary file 5 — Additional file 5: Figure S5. WT and GsdmdI105N/I105N mice retinal RNA profiling and enrichment analysis. A Experimental paradigm with red retinal labelling indicating lesion site. B LogCPM values for raw and normalised counts. C Relative Log Expression plots showing effective normalisation. D Principal component analysis (PCA) identified distinct clustering between groups as visualised in the 3D plot. E Summary of differentially expressed genes with cut-off of adjusted p value < 0.1 and < 0.05 (N = 4–5). [file 12974_2023_2927_MOESM5_ESM.docx]

**Supplementary Figure 5**


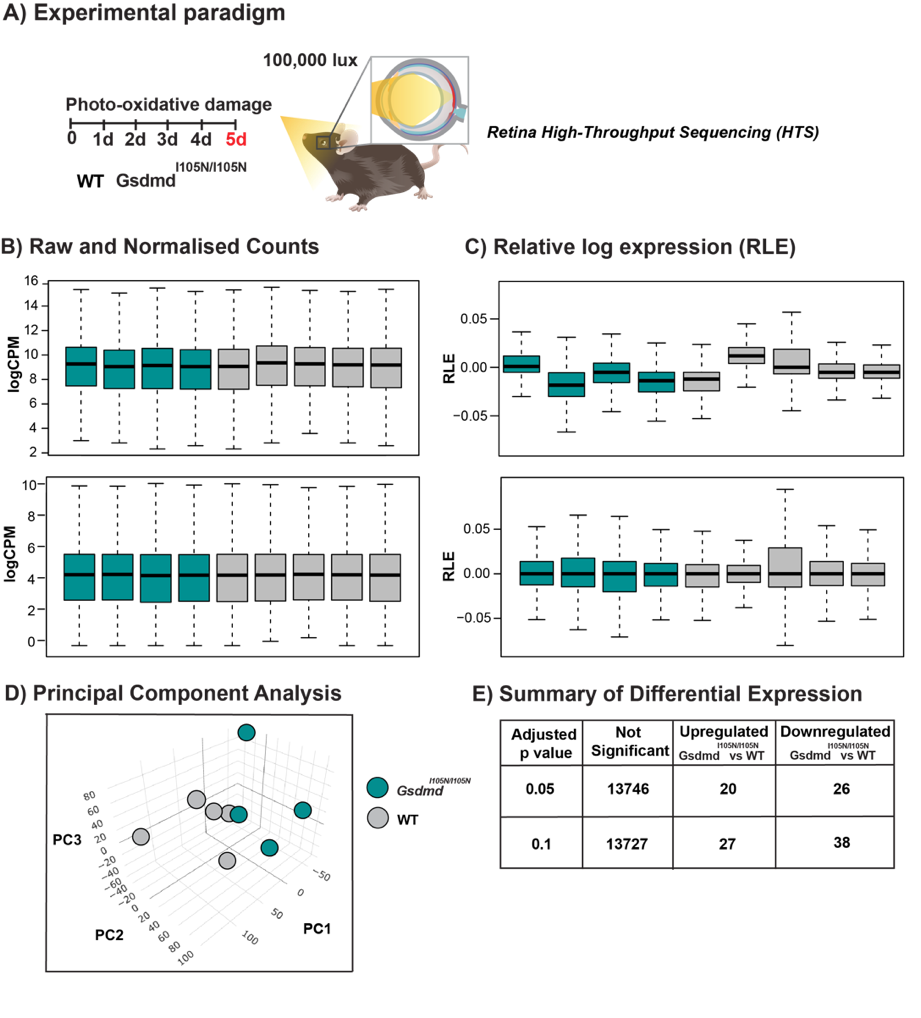


**Supplementary Figure 5: WT and *Gsdmd^I105N/I105N^* mice retinal RNA profiling and enrichment analysis (A)** Experimental paradigm with red retinal labelling indicating lesion site. **(B)** LogCPM values for raw and normalised counts. **(C)** Relative Log Expression plots showing effective normalisation. **(D)** Principal component analysis (PCA) identified distinct clustering between groups as visualised in the 3D plot. **(E)** Summary of differentially expressed genes with cut-off of adjusted p value < 0.1 and <0.05 (N=4-5).
