# Supplementary material for: Impairing Gasdermin D-mediated pyroptosis is protective against retinal degeneration
Source: J Neuroinflammation. 2023 Oct 20;20:239. doi: 10.1186/s12974-023-02927-2 (PMC10588253; doi:10.1186/s12974-023-02927-2)
Supplement: Supplementary file 6 — Additional file 6: Figure S6. WT and GsdmdI105N/I105N mice retinal RNA profiling and differential expression analysis post-PD Heatmaps showing differential expression profile of genes regulating. A Epithelial Mesenchymal Transition (EMT) (adj.p.value <0.1) and B Peroxisome signalling (adj.p.value <0.1). C Scatter plot and heatmap showing no significant change in expression in EV biogenesis and exosome assembly between WT and GsdmdI105N/I105N whole retina post-5-day PD. [file 12974_2023_2927_MOESM6_ESM.docx]

**Supplementary Figure 6**

**
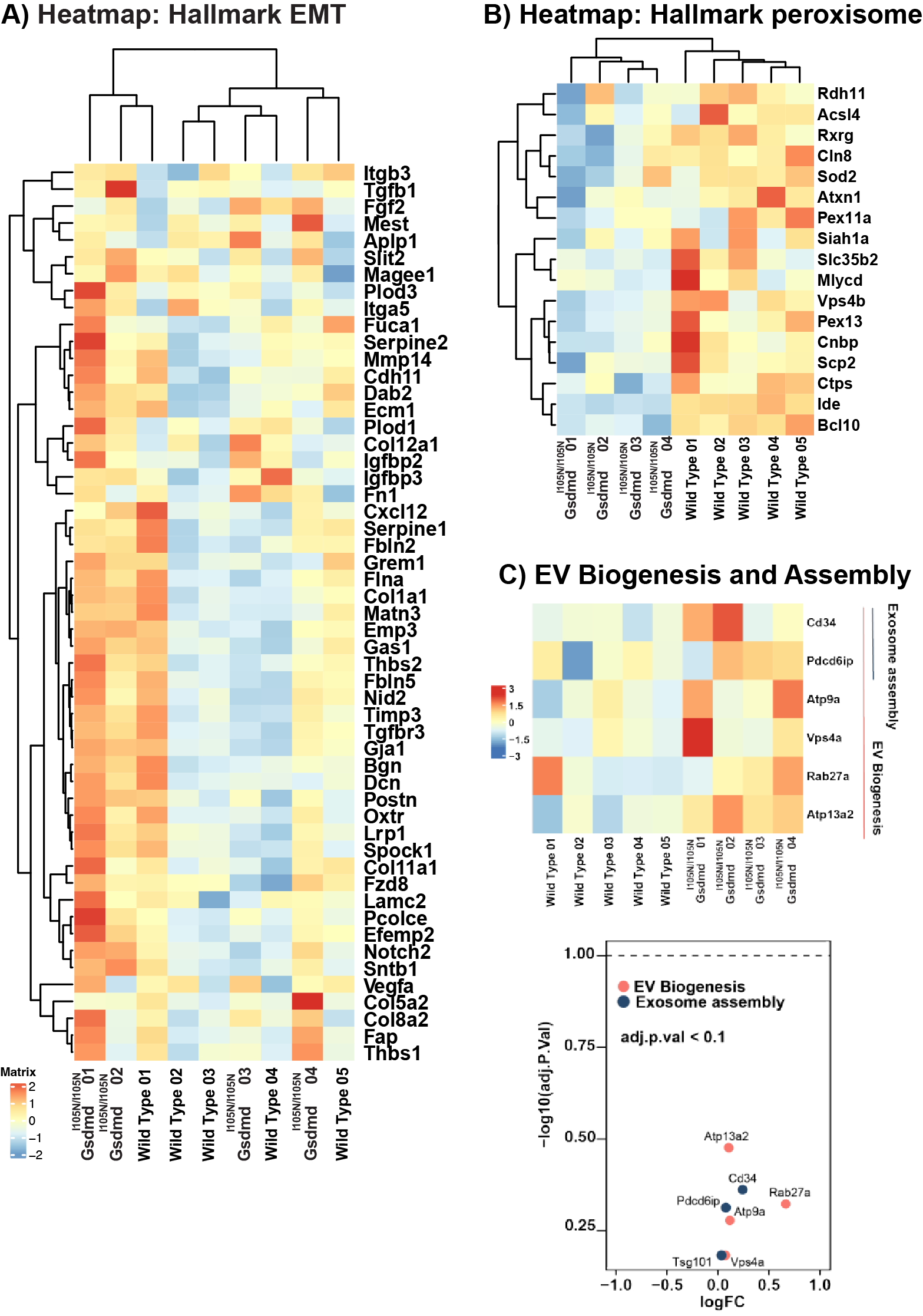
**

**Supplementary Figure 6: WT and *Gsdmd^I105N/I105N^* mice retinal RNA profiling and differential expression analysis post PD.** Heatmaps showing differential expression profile of genes regulating **(A)** Epithelial Mesenchymal Transition (EMT) (adj.p.value <0.1) and **(B)** Peroxisome signalling (adj.p.value <0.1). (C) Scatter plot and heatmap showing no significant change in expression in EV biogenesis and exosome assembly between WT and *Gsdmd^I105N/I105N^* whole retina post 5d PD.
