# Supplementary material for: Impairing Gasdermin D-mediated pyroptosis is protective against retinal degeneration
Source: J Neuroinflammation. 2023 Oct 20;20:239. doi: 10.1186/s12974-023-02927-2 (PMC10588253; doi:10.1186/s12974-023-02927-2)
Supplement: Supplementary file 7 — Additional file 7: Figure S7. iBMDM inhibition using GW4869. A Experimental paradigm showcasing in-vitro EV inhibition followed by inflammasome activation of iBMDM. B Representative, cropped western blots showing bands of 42 kDa for CASP-1, ~50 kDa for full-length GSDMD and ~30 kDa for cleaved GSDMD and 37 kDa for GAPDH reference protein, in iBMDM lysates. C EV concentration measured using NTA in the supernatant of iBMDM with GW4869 compared to DMSO control post-LPS/ATP stimulation, showing a reduction in EV number but no significant change (p > 0.05, N = 4). D Total EV IL-1β release from iBMDM post-LPS/ATP stimulation, as measured by ELISA (p > 0.05, N = 4). [file 12974_2023_2927_MOESM7_ESM.docx]

**Supplementary Figure 7**

**
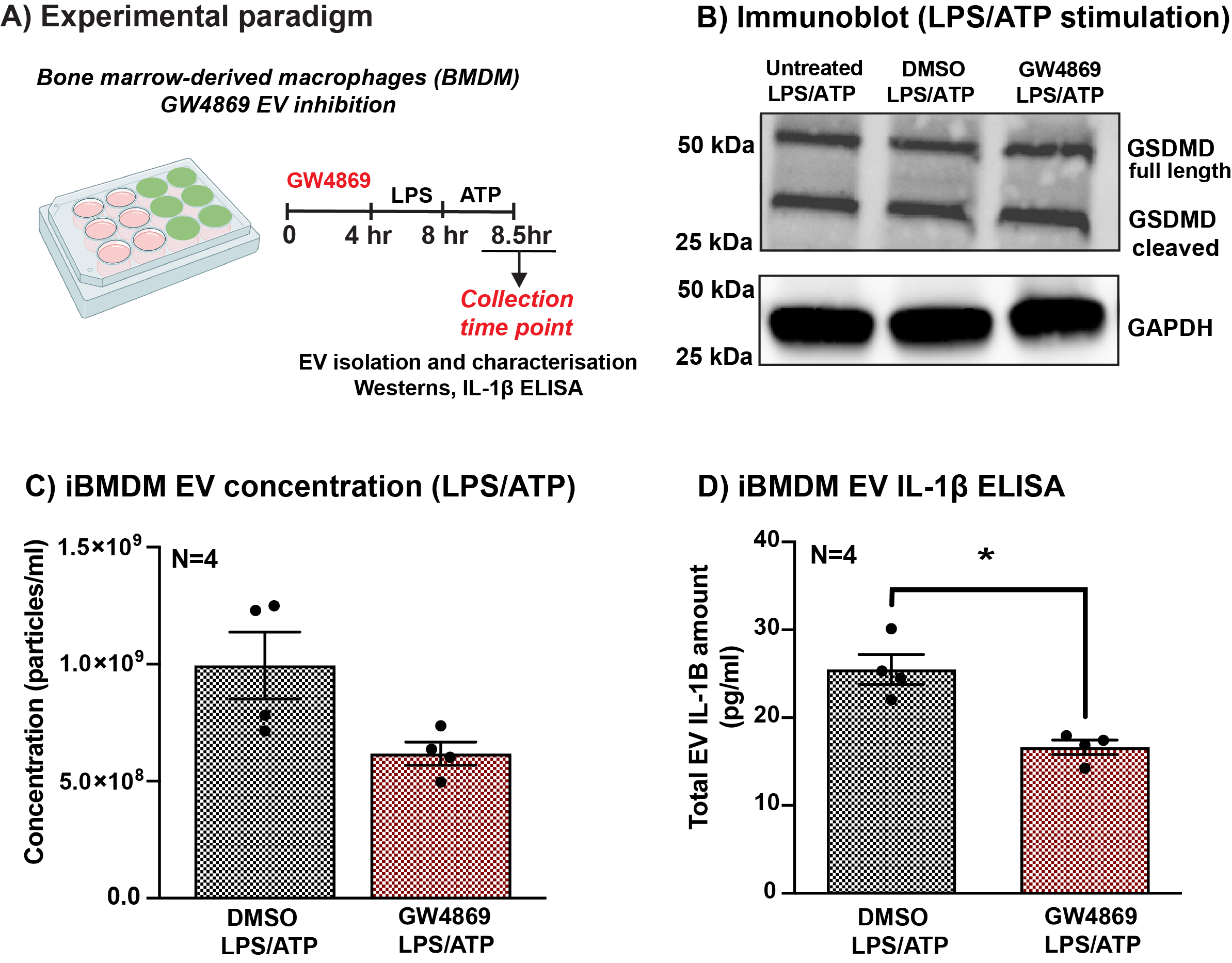
**

**Supplementary Figure 7: iBMDM inhibition using GW4869 (A)** Experimental paradigm showcasing *in-vitro* EV inhibition followed by inflammasome activation of iBMDM **(B)** Representative, cropped western blots showing bands of 42 kDa for CASP-1, ~50 kDa for full-length GSDMD and ~30 kDa for cleaved GSDMD and 37 kDa for GAPDH reference protein, in iBMDM lysates **(C)** EV concentration measured using NTA in the supernatant of iBMDM with GW4869 compared to DMSO control post LPS/ATP stimulation, showing a reduction in EV number but no significant change (p>0.05,N=4) **(D)** Total EV IL-1β release from iBMDM post LPS/ATP stimulation, as measured by ELISA (p<0.05, N=4).
