# Supplementary material for: Impairing Gasdermin D-mediated pyroptosis is protective against retinal degeneration
Source: J Neuroinflammation. 2023 Oct 20;20:239. doi: 10.1186/s12974-023-02927-2 (PMC10588253; doi:10.1186/s12974-023-02927-2)
Supplement: Supplementary file 8 — Additional file 8: Figure S8. Western Blots. A GSDMD Western blot of iBMDM (LPS/ATP) used in Fig. 8B and Additional file 4: Fig. S4B. B CASP-1 Western blot of iBMDM (LPS/ATP) used in Fig. 8B. C GAPDH loading control optimisation blot of iBMDM (LPS/ATP) used in Fig. 8B. D GSDMD and GAPDH western blot of DR retina used in Additional file 1: Fig. S1A. [file 12974_2023_2927_MOESM8_ESM.docx]

**Supplementary Figure 8**

**
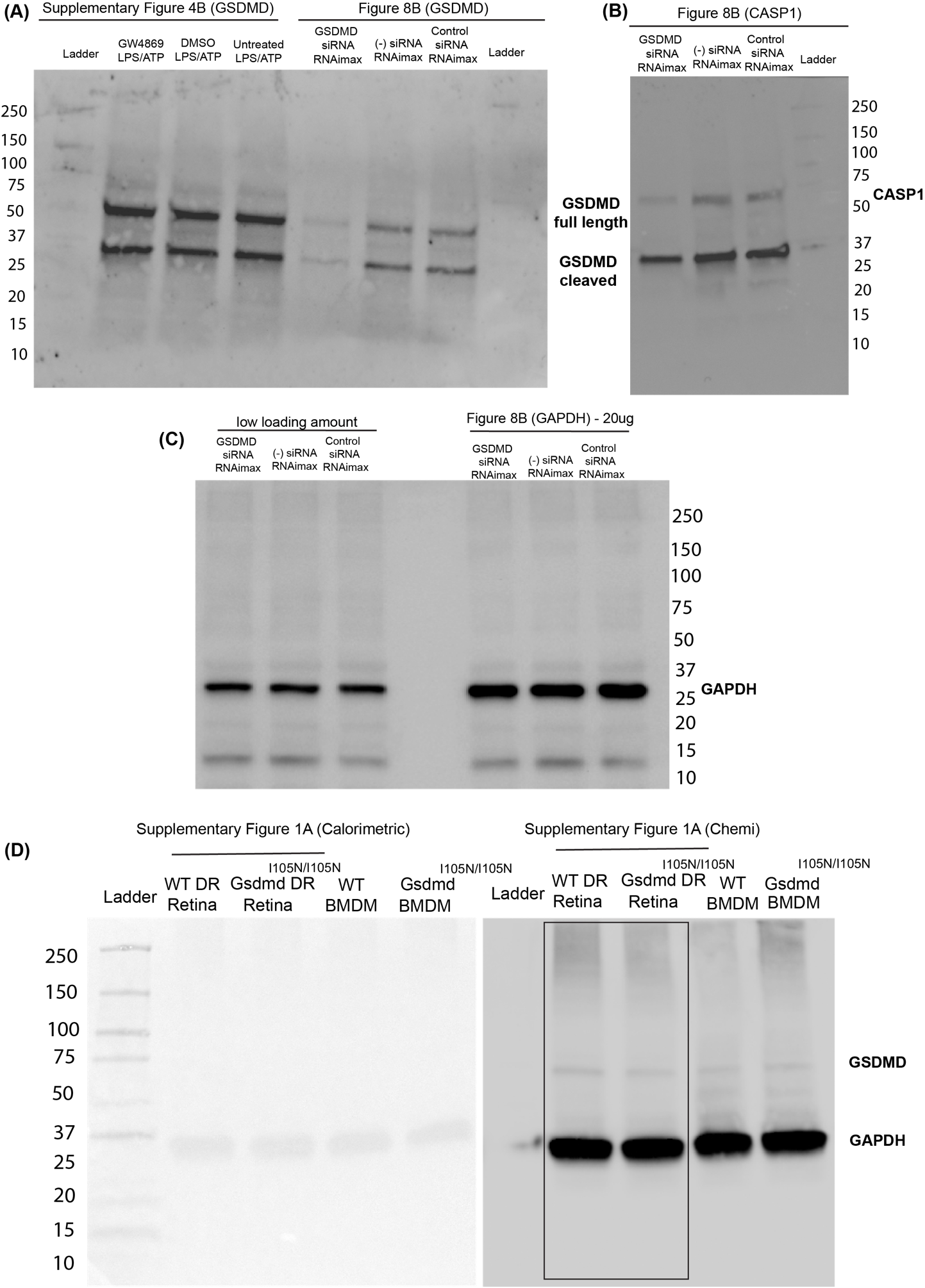
**

**Supplementary Figure 8 : Western Blots (A)** GSDMD Western blot of iBMDM (LPS/ATP) used in Figure 8B and Supplementary Figure 4B (**B)** CASP-1 Western blot of iBMDM (LPS/ATP) used in Figure 8B **(C)** GAPDH loading control optimisation blot of iBMDM (LPS/ATP) used in Figure 8B **(D)** GSDMD and GAPDH western blot of DR retina used in Supplementary Figure 1A
